# Plant-Based, Hydrogel-like Microfibers as an Antioxidant Platform for Skin Burn Healing

**DOI:** 10.1021/acsabm.3c00214

**Published:** 2023-07-26

**Authors:** Fabrizio Fiorentini, Giulia Suarato, Maria Summa, Dalila Miele, Giuseppina Sandri, Rosalia Bertorelli, Athanassia Athanassiou

**Affiliations:** † Smart Materials Group, Istituto Italiano di Tecnologia, Via Morego 30, Genova 16163, Italy; ‡ DIBRIS, Università di Genova, Via Opera Pia 13, Genova 16145, Italy; § Translational Pharmacology, Istituto Italiano di Tecnologia, Via Morego 30, Genova 16163, Italy; ∥ Department of Drug Science, Università di Pavia, Via Taramelli 12, Pavia 27100, Italy

**Keywords:** plant-based microfibers, vitamin C, cross-linking, antioxidant activity, burn healing

## Abstract

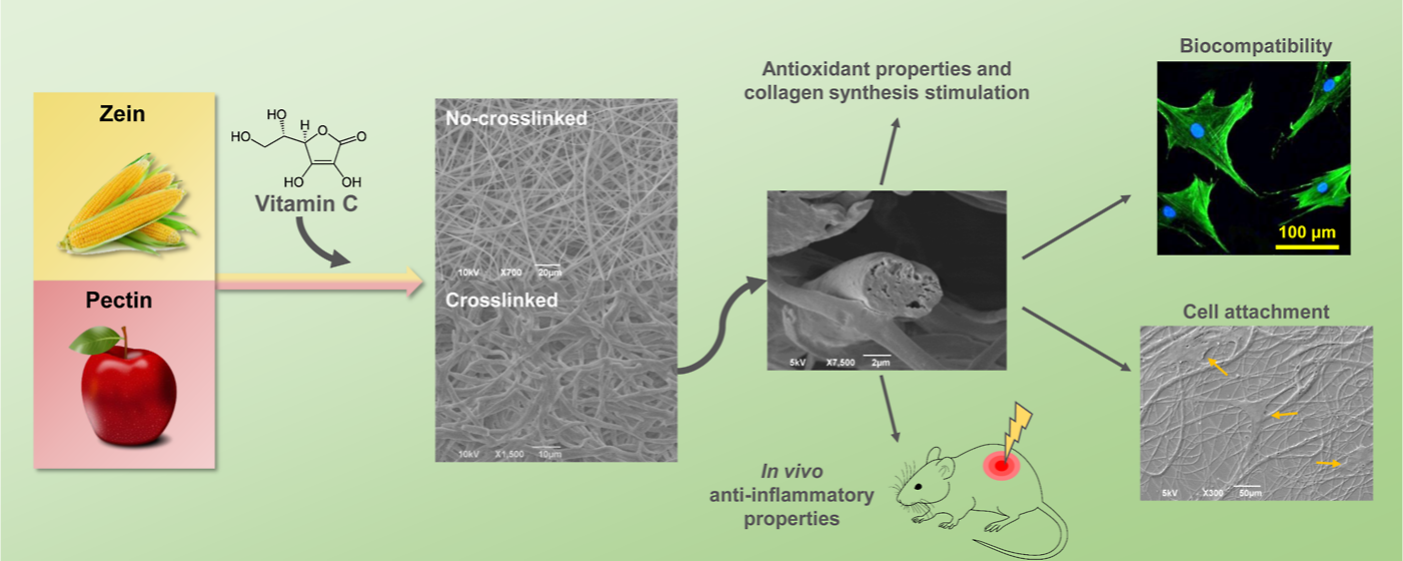

Natural polymers from organic wastes have gained increasing attention in the biomedical field as resourceful second raw materials for the design of biomedical devices which can perform a specific bioactive function and eventually degrade without liberating toxic residues in the surroundings. In this context, patches and bandages, that need to support the skin wound healing process for a short amount of time to be then discarded, certainly constitute good candidates in our quest for a more environmentally friendly management. Here, we propose a plant-based microfibrous scaffold, loaded with vitamin C (VitC), a bioactive molecule which acts as a protecting agent against UV damages and as a wound healing promoter. Fibers were fabricated via electrospinning from various zein/pectin formulations, and subsequently cross-linked in the presence of Ca^2+^ to confer them a hydrogel-like behavior, which we exploited to tune both the drug release profile and the scaffold degradation. A comprehensive characterization of the physico-chemical properties of the zein/pectin/VitC scaffolds, either pristine or cross-linked, has been carried out, together with the bioactivity assessment with two representative skin cell populations (human dermal fibroblast cells and skin keratinocytes, HaCaT cells). Interestingly, col-1a gene expression of dermal fibroblasts increased after 3 days of growth in the presence of the microfiber extraction media, indicating that the released VitC was able to stimulate collagen mRNA production overtime. Antioxidant activity was analyzed on HaCaT cells via DCFH-DA assay, highlighting a fluorescence intensity decrease proportional to the amount of loaded VitC (down to 50 and 30%), confirming the protective effect of the matrices against oxidative stress. Finally, the most performing samples were selected for the in vivo test on a skin UVB-burn mouse model, where our constructs demonstrated to significantly reduce the inflammatory cytokines expression in the injured area (50% lower than the control), thus constituting a promising, environmentally sustainable alternative to skin patches.

## 1 Introduction

Wound healing depends on high coordination among essential mediators, such as cytokines, inflammatory cells, growth factors, proteinase, and extracellular matrix (ECM).^1^ In addition, the role of reactive oxygen species (ROS) is pivotal in the wound healing process. At low concentrations, ROS possess a positive influence in the injured area, such as mediation of the vasoconstriction, lymphocytes recruitment, defiance against pathogens, and tissue repair. On the contrary, when highly present in the wound bed, ROS species lead to oxidative stress, which hinders the lesion repair, resulting in the establishment of a chronic condition.^2,3^ For this reason, ROS modulation represents a crucial step to promote the wound healing process.^4^ Although cells such as keratinocytes and fibroblasts play a vital role in restoring skin functions, the ECM itself provides the essential substrate for their interaction. Indeed, the ECM consists of an intricate three-dimensional scaffold composed of proteins and proteoglycans, extremely important during tissue restoration due to its ability to modulate cell behavior and tune the secretion of proteases and growth factors.^5^ Among the main ECM components are the collagens, a large family of triple-helical proteins that are ubiquitous in the human body and are involved in a broad range of functions, such as cell adhesion, cell migration, and tissue repair.^6,7^ More specifically, collagen III and collagen I play an important role in the wound healing as the first one is involved in the primary phases of the process, while the second one is mostly deposited in the re-epithelization step.^8^ Wound dressings able to stimulate collagen synthesis have been shown an improvement in the overall tissue healing.^9,10^

Nowadays, a wide range of techniques can be exploited for the fabrication of different types of micro- and nanostructured, advanced wound dressings (e.g., films, hydrogels, composite membranes, and fibers).^11^ Compared to the conventional ones, such as patches and gauzes, these smart dressings can offer good protection against the insurgence of microorganism-driven infections and from chemical/physical external aggressions, while promoting the healing process via stimulation of cell adhesion and proliferation. Electrospinning represents an effective approach for the fabrication of nano- and microfibrous scaffolds.^12^ The high-surface area, the three-dimensionality of the fibrous network, and the structural and topographical similarities with the ECM make electrospun-prepared matrices optimal candidates for skin tissue restoration.^13^

Natural polymers have been widely used to fabricate fibrous mats^14^ and, due to their controlled biodegradability and biocompatibility, plant-based polymers may offer several benefits as building blocks for tissue engineering scaffolds.^15^ Furthermore, these materials are very abundant as organic wastes which are regularly discarded and no longer used. The valorization of waste biomaterial products has received more and more attention in various fields of applications, including the biomedical one.^16−18^ Due to their high availability and their cheap market price, some biowaste-derived polymers hold very promising features for wound care-related strategies.^19^ In general, biomaterials obtained from leftovers of various industrial processes (i.e., agriculture, textile, aquaculture, and food production) are considered as green options to diminish environmental pollution and waste formation and disposal, thus positively contributing to a circular economy of the resources.^20^ In this regard, plant-based biopolymers represent one of the main sources retrievable from agro-industrial biomass residues, which are then re-introduced in the production chain for applications in cosmetics, nutraceuticals, and pharmaceutical industry as scaffolding materials, active fillers, or drug delivery systems.^21^

Zein is a biodegradable plant-based protein obtained from an abundant renewable agricultural source, corn.^22^ Moreover, considering that the corn market is massively widespread around the World with an annual production of over 1 billion tons, zein becomes readily available in the environment as organic waste.^23^ Over the past few decades, zein has been processed in several shapes such as nano/microparticles, nano/microcapsules, nanofibers, films, and hydrogels, thus showing a high versatility in acquiring specific morphologies for the delivery of bioactive agents.^24−26^ Cui and co-workers successfully fabricated co-electrospun PVA/zein nanofibers loaded with a flavonoid molecule, demonstrating their ability to promote fibroblast proliferation in vitro.^24^ In another study, a zein bilayer was designed for the controlled release of gentamicin to prevent wound infections, showing a sustained delivery of the antibiotic molecules in the early phase of the treatment.^25^ With the aim of improving the antibacterial potential of zein, nanofibers were also electrospun with Ag nanocomposites.^26^ Babitha and Korrapati fabricated zein-polydopamine polymeric scaffolds impregnated with TiO_2_ nanoparticles and showed their capacity to stimulate in vitro cell migration and promote wound closure after 15 days of treatment in an in vivo full-thickness dermal excisions murine model.^27^

Nowadays, commercially pectin is extracted mostly from waste materials of citrus fruits (e.g., orange, apple, lemon, and grapefruit), in fact, it is estimated that dried citrus peel contains approximately 30% of pectin, while dried apple pulp about 20%.^28^ Pectin possesses the ability to gel through a mechanism known as the “egg-box” model, a Ca-dependent gelation in which the polysaccharide chains form egg-box dimers with Ca^2+^ ions, generating multimers.^29^ In the last years, potential uses of pectin in the biomedical field as a drug delivery carrier or as a scaffold material for the treatment of damaged skin, in the form of electrospun fibers,^30^ films,^31^ and hydrogels, have been investigated.^32−34^ Pectin natural properties confer numerous advantages, such as hydroscopicity, which promotes the wound exudate removal and the ability to inhibit bacterial growth.^35^ Lin et al. demonstrated how electrospun nanofibers of pectin and chitosan were able to stimulate the cell proliferation of fibroblast and the secretion of type I collagen, important for tissue regeneration.^30^ Furthermore, an in vivo full-thickness excisional lesion model treated with pectin hydrogels has shown the ability of this plant-based material to speed up the healing process.^33^

The complex tissue healing process requires the interplay of a variety of bioactive molecules and cellular cohorts to be as much effective as possible.^36,37^ Among the bioactive factors, ascorbic acid, more commonly known as vitamin C (VitC)—an acidic, water-soluble antioxidant—is a co-factor of several enzymes which humans are unable to synthesize.^38,39^ VitC is known to possess abilities to neutralize free radicals and to act as skin-protecting agent against UV-induced damages and some skin carcinomas. For example, a dose of 60–640 mg of VitC per gram of epidermis and a dose of 30–130 mg/g of dermis have been reported to counteract photodamage.^40^ However, this molecule is chemically unstable and easily prone to oxidation itself; therefore, strategies involving the synthesis of more stable derivatives as well as the design of topical formulations are needed to efficiently pass the thick and hydrophobic stratum corneum (which usually repels water-soluble molecules) and increase the skin penetration.^41^ To this end, several approaches have been proposed to protect this molecule from degradation and to target its delivery, such as creams, liposomes, and polymeric nanoparticles.

Nevertheless, VitC is a relevant precursor in collagen synthesis,^42^ and a gradual and dense secretion of collagen fibers were seen for fibroblasts in the presence of VitC.^43^ Moreover, Lima et al. conducted a study on wound healing in rats, topically applying a cream containing 10% VitC, demonstrating a consistent acceleration of the healing in the treatment group and a decline of macrophage number, highlighting the molecule anti-inflammatory action. In a study by Yun and co-workers, topical application of VitC in a silicone gel resulted in a significant reduction of permanent scar formation.^44^

In this study, zein and pectin were exploited for the fabrication of biocompatible and biodegradable microfibers entirely composed of plant-based molecules, for the treatment of skin burns, a condition in which the balance between the ROS and the antioxidant agents can be drastically affected when burn occurs on the skin, resulting in cell and tissue damage.^45^ The microfibers were fabricated through vertical electrospinning of liquid suspensions and were loaded with different concentrations of the antioxidant VitC. Subsequently, the samples were cross-linked to stabilize the structure of the scaffold and to tune the release of the bioactive molecule. The samples were thoroughly characterized in terms of their chemical and physical properties and biocompatibility toward human dermal fibroblast adult (HDFa) cells and human keratinocytes (HaCaT). More interestingly, the radical scavenging activity of VitC was monitored, both in a tube test and on keratinocytes in vitro, further confirming that our microfibers were effective in preserving its antioxidant proprieties. The ability of the released VitC to stimulate the synthesis of collagen I and collagen III was monitored through real-time Rt-qPCR onto mRNA samples extracted from HDFa cells. A mild (UVB) burn murine model was exploited to evaluate the ability of our designed dressing to tackle skin inflammation.

## 2 Materials and Methods

### 2.1 Materials

Zein powder (MW 20 kDa), ethanol (≥99.7%), l-ascorbic acid (Vit C), phosphate-buffered saline (PBS) 1×, sodium acetate, calcium chloride (CaCl_2_), apramycin sulfate salt, trifluoroacetic acid (TFA), and ethanol/hexamethyldisilizane (36%) were purchased from Sigma-Aldrich and used as received. Low methoxy pectin powder was purchased from Silva Team. Lecithin (90%) soybean was purchased from Alfa Aesar. The cell proliferation reagent [3-(4,5-dimethylthiazol-2-yl)-5-(3-carboxymethoxyphenyl)-2-(4-sulphophenyl)-2*H*-tetrazolium] (MTS) and the CellTiter-Glo Luminescent viability assay kit were obtained from Promega. HDFa, fibroblast basal medium supplemented with Supplement Pack Fibroblast Growth Medium 2, 0.25% trypsin–EDTA (1×), 2-(4-aminiodinophenyl)-6-idolecarbamide dihydrochloride (DAPI), and Alexa Fluor 488 Phalloidin were purchased from Thermo Fisher Scientific. HaCaT cells were purchased from the Cell Line Service (Heidelberg, Germany).

### 2.2 Fabrication of the Microfibers and Cross-Linking Strategies

Zein, pectin, and soy lecithin were mixed in an ethanol solution (80% v/v) at fixed concentrations of 40, 3, and 5% (w/v), respectively. VitC was added at different concentrations (0.17, 1.70, 3.40, and 10.00 mg/mL). Subsequently, the solutions were sonicated with a probe sonicator (Sonic Vibra-Cell, 750 W, 20 kHz) for 1 min with 40% amplitude. The process was performed following the scheme of 15 s on/15 s off, to obtain the various suspensions needed for the electrospinning process. The electrospinning solutions were loaded into a 10 mL syringe equipped with an 18-gauge stainless-steel needle. The microfibers were obtained by means of an electrospinning setup comprising a syringe pump (NE-1000, New Era Pump Systems, Inc.), working at a constant flow rate of 2.0 mL/h and with an electric potential set at 20 kV. The microfiber mats were collected on an aluminum sheet covering a collector plate. The distance between the tip of the needle and the surface of the Al foil used as the collector was kept at 25 cm. The electrospinning process was carried out under ambient conditions (21 °C, with a relative humidity of 50%). The whole process is schematized in Figure S1. The obtained samples, with generic name ZPCs, were labeled as ZPC0, ZPC1, ZPC2, ZPC3, ZPC4, and ZPC5, as reported in Table 1.

**Table 1:** Composition of ZPCs Samples

samples	ZPC0	ZPC1	ZPC2	ZPC3	ZPC4
zein (mg/mL)	400				
soy lecithin (mg/mL)	50				
pectin (mg/mL)	30				
VitC (mg/mL)	0	0.17	1.7	3.4	10

Three cross-linking (CL) strategies were preliminary tested onto the composite, unloaded matrices ZPC0: (1) immersion of the sample in a CaCl_2_ solution (15% w/v) for 5 min at room temperature (RT), followed by two washing steps in PBS; (2) exposition to a TFA-saturated environment for 3 min, followed by two washing steps in PBS; and (3) exposition to a TFA-saturated environment for 3 min, without washing. The best strategy was selected through morphological analysis, diameter evaluation, and a preliminary biocompatibility test on HDFa cells. The cross-linked samples were labeled as ZPC0_CL_, ZPC1_CL_, ZPC2_CL_, ZPC3_CL_, and ZPC4_CL_.

### 2.3 Morphological Tests

The top-view morphology and the cross-section of the microfibers were analyzed by JEOL JSM-6490LA scanning electron microscopy (SEM), with an acceleration voltage of 10 kV. Before observation, the double-side adhesive carbon tape was placed on an aluminum stub, to accommodate small pieces of the microfibers, which were subsequently sputter-coated with a thin layer of gold (10 nm) under high vacuum conditions.

For the cross-section inspection, ZPC0 and ZPC0_CL_ microfibers were immersed in liquid nitrogen for a few minutes and, subsequently, freeze-dried for 2 days (Christ, Epsilon 2–4 LSCplus, air refrigeration). The resulting samples were manually cut with a cutter after another wash in liquid nitrogen, in order to expose the cross-section for image analysis.

### 2.4 Fourier Transform Infrared

The chemical analysis of the microfibers was performed by attenuated total reflectance (ATR) accessory (MIRacle, ATR, PIKE Technologies) coupled to a Fourier transform infrared (FTIR) spectrometer (Equinox 70 FT-IR, Bruker). All spectra were reordered over a range of 4000–600 cm^–1^, with 4 cm^–1^ resolution (accumulating 128 scans).

### 2.5 Vitamin C Release and DPPH^•^ Assay

VitC release assay was performed both on the non-cross-linked microfibers and on the cross-linked ones. From each sample, three pieces of 4 cm^2^ were cut and incubated in 3 mL of PBS (pH 7.4), in an oven set at 37 °C. At each time point (15, 30, 60, 180, 540, and 1440 min), 3 mL of PBS was collected and replaced with 3 mL of fresh PBS buffer. The absorbance of the samples was evaluated using a CARY 300 Scan UV–visible spectrophotometer, considering the molecule characteristic peak at 260 nm. The cumulative amount of the bioactive molecule released in PBS was evaluated by comparing the data obtained with a predetermined calibration curve. For each sample, triplicates were considered. In parallel, the same assay was performed for the cross-linked microfibers. The treatment was performed by immersing three pieces of 4 cm^2^ of each microfibrous mat in an aqueous solution of CaCl_2_ 15% (w/v in MilliQ water) for 5 min at RT. Afterward, two washing steps with PBS were carried out to remove the residual salt, and the experiment was carried out as described above.

The DPPH^**•**^ assay was performed to evaluate the antioxidant activity of the VitC released from the non-cross-linked and cross-linked microfibers, after their immersion in 3 mL of PBS for specific time points (15, 30, 60, 180, 540, and 1440 min), in an oven set at 37 °C. An aliquot of each extract solution (1.0 mL) was added to 2.0 mL of 0.2 mM solution of DPPH^**•**^ radical reagent in pure ethanol. To avoid light degradation, the experiment was performed in the dark. After 1 h of reaction, the absorbance (*A*_1_) was determined at 517 nm by an UV–vis spectrophotometer. Another absorbance value (*A*_2_) was obtained from 1.0 mL aliquots of each extract solution mixed with 2.0 mL of ethanol. Meanwhile, a control absorbance value (*A*_3_) was measured from a mixture of 2.0 mL of a 0.2 mM DPPH^**•**^ free-radical reagent solution in ethanol. The percentage of DPPH^**•**^ free-radical scavenging activity was calculated following eq 1
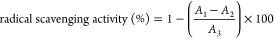
1

### 2.6 Degradation and Swelling Assay

The ability of the microfibers to uptake water and their tendency to undergo degradation in an aqueous environment was tested on non-cross-linked and cross-linked ZPC0 matrices. Several pieces of 1.4 cm of diameter were previously weighted, and half of those were cross-linked (see paragraph 2.2). All the samples were immersed in 3 mL of PBS solution (pH 7.4) or PBS supplemented with Protease XIV from *Streptomyces griseus* (≥3.5 units/mg, Sigma-Aldrich). Apramycin sulfate salt (0.01% w/v) was added to the solution to prevent bacterial contamination. At each time point (1, 2, 3, 5, and 7 days), samples were retrieved, and the water in excess was removed from the surface. Subsequently, the samples were washed trice for 1 min each in pure water, dried, and after that they were weighed. The percentage of swelling capacity (%) of the various materials was calculated from eq 2
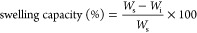
2where *W*_s_ and *W*_i_ are the swollen weight and the initial weight of each sample, respectively. Both tests were conducted in triplicates, with three independent experiments.

A degradation study was performed under the same conditions as the water uptake test. Before the weight measurement, samples were taken out from the incubation buffers and then dried at RT for 48 h. The weight loss (%) was calculated through eq 3, as follows
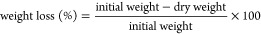
3

Possible morphology variations were monitored by observing the ZPC0 microfibers at the scanning electron microscope after 1 and 7 days of immersion in the various buffers. The percentage of swelling and degradation was analyzed considering triplicates for each group.

### 2.7 Biocompatibility Assay and Cell Morphology Analysis

HDFa cells were used to investigate the in vitro biocompatibility of the ZPCs microfibers. Cells were cultured in T75 culture flasks with fibroblast basal medium supplemented with Supplement Pack Fibroblast Growth Medium 2 in a humidified incubator at 37 °C and with 5% CO_2_. At a confluence of approximately 80%, the cells were trypsinized and seeded onto 24-well plates at a density of 5000 cells/cm^2^ in 0.5 mL of medium. Simultaneously, an extraction medium from the samples was prepared following the procedure described in the ISO10993-5 standard test. The microfibers were sterilized for 30 min (15 min for each side) under ultraviolet (UV) light. Afterward, 6 cm^2^ of the samples was immersed in 1 mL of cell culture medium for 24 h at 37 °C. After 24 h of culture, the medium was replaced with the extraction one, and the cells were incubated for a further 24, 48, and 72 h. The cell viability was determined by MTS assay, a colorimetric method for sensitive quantification of viable cells. The NAD(P)H-dependent dehydrogenase enzymes in metabolically active cells cause the reduction of the MTS tetrazolium compound and generate a colored formazan product that is soluble in the cell culture medium. This difference can be quantified by measuring the absorbance at 490 nm.

Biocompatibility was also tested with primary human keratinocytes (HaCaT). Cells were cultured in DMEM supplemented with 10% fetal bovine serum and 2 mmol/L of l-glutamine at 37 °C in an atmosphere of 5% CO_2_ and 95% air. Cell viability was conducted using CellTiter-Glo Luminescent viability assay (Promega, MI, Italy). HaCaT cells were seeded in 96-well plates at a density of 3.5 × 10^5^ and incubated until the proper confluence was reached. After 24 h of treatment, cells were rapidly rinsed with pre-warmed PBS with Ca^2+^/Mg^2+^, the medium was replaced with the extraction one (control samples were treated with medium processed as the extractions), and cells were incubated for an additional 24 and 48 h. Extracts were prepared by placing the zein fibers into the cell medium at different concentrations. According to ISO10993-5 guidelines, all the extraction materials were considered biocompatible if the final cell viability of the sample was higher than 70% of the control group. Viability was determined by measuring ATP levels by CellTiter-Glo assay, as indicated by the supplier as percentage survival relative to control cells. Data represent mean ± SD of three independent experiments.

To investigate the cell morphology, HDFa cells were grown onto 13 mm coverslips and incubated for 24 h either with fibroblast basal medium or with the matrices extraction medium, for the control and treated samples, respectively. After the incubation period, the medium was removed, and cells were washed with fresh pre-warmed PBS and fixed with 3.7% paraformaldehyde (PFA) in PBS for 15 min. A DAPI solution (2.5 μg/mL) was applied for 15 min in the dark for the nuclei staining. For the actin fibers staining, samples were first permeabilized with 0.3% Triton X-100 for 8 min and washed two times with PBS. Alexa Fluor 546 solution, diluted 1:100 in PBS, was added to each well and incubated for 20 min at RT, covered with Al foil. Subsequently, the cells were washed trice with PBS and then mounted directly on cover glasses (via Fluoromount-G) for confocal inspection. Cell imaging was carried out via the confocal microscope Nikon A1 equipped with 405 and 488 nm lasers, and images were taken with 20× magnification.

### 2.8 Direct Plating of Primary Fibroblasts onto the Composite Fibrous Matrices

HDFa cells were cultured in T75 culture flasks with fibroblast basal medium supplemented with Supplement Pack Fibroblast Growth Medium 2 in a humidified incubator at 37 °C and with 5% CO_2_. At a confluence of approximately 80%, the cells were trypsinized and seeded directly on the ZPC0 and ZPC3 microfibrous mats at a density of 5000 cells/cm^2^ in 0.5 mL of medium. The fibrous samples were electrospun directly on 13 mm glass coverslips, able to fit inside the wells of a 24 well-plate. The microfibers were previously sterilized for 30 min (15 min for each side) under ultraviolet (UV) light. Pristine glass coverslips were used as controls. After 72 h of growth, HDFa cells were fixed in a solution of 2% glutaraldehyde in 0.1 M cacodylate buffers for 2 h, at RT. Subsequently, the samples were post-fixed with osmium tetroxide (1% in water) for 2 h and washed with MilliQ water. After that, the microfibers were dehydrated with a series of incubations in increasing concentrations of ethanol in water solutions (from 30 to 100%, 10 min each), followed by incubation in 1:1 ethanol/hexamethyldisilazane (HDMS) and 100% HMDS. Lastly, the samples were dried overnight in air and then sputtered with a 10 nm gold layer. Imaging analysis was performed using a JEOL JSM-6490LA scanning electron microscope equipped with a tungsten filament and operating at 10 kV of accelerating voltage.

### 2.9 In Vitro Antioxidant Assay onto Human Keratinocytes (HaCaT Cells)

To assess ROS generation by fluorimeter analysis, HaCaT cells were plated in a 96-well optical bottom white microplate at a density of 5 × 10^5^ in a final medium volume of 100 μL. After 24 h of cell growth in the presence of the cross-linked and no-cross-linked microfibers extraction medium, cells were incubated with 1 mM dichlorofluorescein diacetate (DCFH-DA) in PBS for 45 min at 37 °C in the dark. Cells treated only with H_2_O_2_ were used as positive control, and VitC at a concentration of 10 mg/mL (the higher amount of the active molecule loaded inside the microfibrous samples prepared in this study) was used as additional control. The presence of per-oxides due to the oxidative burst in the cells could be monitored by the analysis of the conversion of non-fluorescent DCFH-DA to the highly fluorescent compound 20,70-dichlorofluorescein (DCF) by cellular esterase. The emitted fluorescence is directly proportional to the concentration of hydrogen peroxide inside the cell. After washing in PBS, the cells were analyzed with a fluorimeter (microplate reader Tekan): the excitation filter was set at 485 nm, and the emission filter was set at 535 nm.

### 2.10 Rt-qPCR Analysis for Gene Expression

Rt-qPCR was performed to investigate the in vitro distinct differences in expression of the ECM components and the proliferation/apoptotic stimuli of fibroblasts after 24 h and 7 days of contact with non-cross-linked and cross-linked microfibers. Collagen type I (*Col*-1a), collagen type III (*Col*-3a), *bcl*-2, and *bax* genes expressions were evaluated. Rt-qPCR was conducted on a total of three samples per time point, and cells treated in standard conditions were employed as control. The total RNAs were isolated with the TriZol agent (Thermo Fisher Scientific, Italy) according to the manufacturer’s instructions and quantified spectrophotometrically at 230 nm by means of a FLUOstar Omega microplate reader (FLUOstar Omega—BMG LabTech, D) equipped with a L-vis microplate. 1 μg of RNA was used as template for the synthesis of the cDNA, and reverse transcription was carried out using the SimpliAmp Thermal Cycler, following the manufacturer’s instructions of the iScript cDNA Synthesis Kit (BioRad, Milan, Italy). Primers for detecting gene expression of *Col-*1a (Unique Assay ID: qHsaCED0002181), *Col-*3a (Unique Assay ID: qHsaCED0046560), *bax* (Unique Assay ID: qHsaCED0037943), and *bcl*-2 (Unique Assay ID: qHsaCED0004655) were designed by Biorad (Biorad, Milan, Italy). PCR solution (20 μL) was composed of 1 μL of cDNA (25 ng), 10 μL of master mix solution of SsoAdvanced Universal SYBR Green Supermix (Biorad, Milan, Italy), and 1 μL at 250 nM of each primer. The ΔΔ*Ct* method was used for the data analysis, and the gene expressions were normalized for the housekeeping gene GAPDH. The sequences and the amplicon length of the primers involved in the study are listed in Table 2. The thermal cycling program was performed by means of a StepOnePlus Real-Time PCR System and set as follows: polymerase was activated in 30 s at 95 °C, subsequently the DNA denaturation was reached in 15 s at 95 °C and the annealing step at 60 °C for 30 s. Denaturation and annealing cycles were repeated 40 times. Finally, melt curves were recorded.

**Table 2:** List of the Primer Acquired and Relative Gene Sequence Description

primer	abbreviation	amplicon context sequence	amplicon length (bp)
collagen, type I, alpha 1	COL1A1	CCCCCGCATGGGTCTTCAAGCAAGTGGACCAAGCTTCCTTTTTTAAAAAGTTATTTATTTATTCTTTTTTTTTTTTTTTTTTTGGTAAGGTTGAATGCACTTTTGGTTTTTGGTCATGTTCGGTTGGTCAAAGATAAAAACTAA	113
collagen, type III, alpha 1	COL3A1	ACACCGATGAGATTATGACTTCACTCAAGTCTGTTAATGGACAAATAGAAAGCCTCATTAGTCCTGATGGTTCTCGTAAAAACCCCGCTAGAAACTGCAGAGACCTGAAATTCTGCCATCCTGAACTCAAGAGTGGA	90
B-cell CLL/lymphoma 2	BCL2	TTGGACGAGGGGGTGTCTTCAATCACGCGGAACACTTGATTCTGGTGTTTCCCCCTTGGCATGAGATGCAGGAAATTTTTATTCCAATTCCTTTCGGATCTTTATTTCATGAGGCACGTTATTATTAGTAAGTATTGTTAATATCAGTCTACTTCCTCTGTGATGCTGAAAGGTT	145
BCL2-associated X protein	BAX	GCACCAAGGTGCCGGAACTGATCAGAACCATCATGGGCTGGACATTGGACTTCCTCCGGGAGCGGCTGTTGGGCTGGATCCAAGACCAGGGTGGTTGGGTG	71
glyceraldehyde-3-phosphate dehydrogenase	GAPDH	GTATGACAACGAATTTGGCTACAGCAACAGGGTGGTGGACCTCATGGCCCACATGGCCTCCAAGGAGTAAGACCCCTGGACCACCAGCCCCAGCAAGAGCACAAGAGGAAGAGAGAGACCCTCACTGCTGGGGAGTCCCTGCCACAC	117

### 2.11 In Vivo Studies on UVB-Induced Skin Inflammation

8 weeks old male C57BL/6J mice (Charles River, Calco, Italy) were used for the study. Animals were grouped in ventilated cages and were able to freely access food and water. They were maintained under controlled conditions: temperature (21 ± 2 °C), humidity (50 ± 10%), and light (10 and 14 h of light and dark, respectively). All animal experiments were performed according to the guidelines established by the European Communities Council Directive (Directive 2010/63/EU of 22 September 2010) and approved by the National Council on Animal Care of the Italian Ministry of Health. All efforts were made to minimize animal suffering and use the lowest possible number of animals required to produce statistically relevant results, according to the “3Rs concept”. Animals were anesthetized with a mixture of ketamine (10%) and xylazine (5%), which was administered via a single intraperitoneal injection before inducing UVB inflammation. Animal dorsal skin was shaved with an electric clipper, and the burn wounds were induced, as reported in the literature. Briefly, mice were placed in a tube of UV opaque material with a squared opening of approximately 1.5 cm^2^ in the desired portion of skin and exposed to a narrowband UVB light source (TL01 fluorescent tubes, Philips, UK, λ_max_ = 312 nm) able to produce an even field of irradiation (maximal dose of 1000 mJ/cm^2^). Following burn induction, the exposed area was immediately treated by placing ZPC3 and ZPC3_CL_ patches and covered with Tegaderm in order to prevent the mice from removing the treatment. The SHAM group was also covered with Tegaderm. Naïve mice followed the same procedures without being exposed to UVB radiation and without any pharmacological treatment. Animals were sacrificed at 48 h post UVB burn induction, and samples from UVB-exposed and non-exposed skins were removed and stored at −80 °C until processing. Each sample was homogenized, subsequently centrifuged, and the supernatant isolated and stored at −80 °C. The expression of cytokines IL-6, IL1β, and TNF-α was measured using ELISA quantikine kit (R&D system), according to the manufacturer’s instructions.

### 2.12 Statistical Analysis

All the experiments were performed in triplicates, and the results were reported as mean ± standard error. The statistical significance was evaluated using one-way ANOVA, followed by Bonferroni’s post hoc test using Origin 2018 64Bit. Values of *p* < 0.05 (*), *p* < 0.01 (**), and *p* < 0.005 (***) were considered significant.

## 3 Results and Discussion

### 3.1 Morphological Inspection and Chemical Characterization of the Composite Microfibers

**Figure 1:**
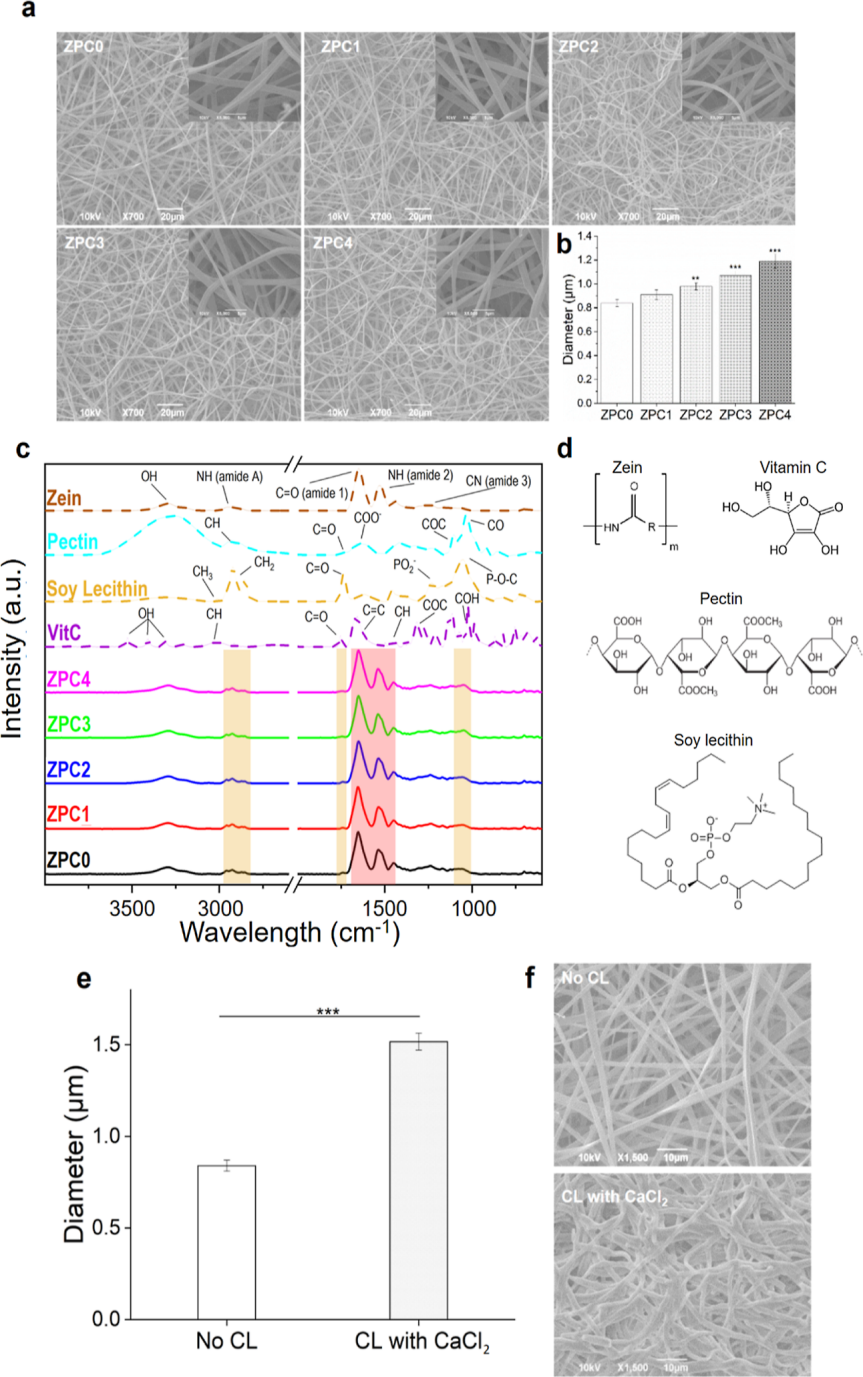
Physical-chemical characterization of the composite fibers. (a) SEM images of ZPC0, ZPC1, ZPC2, ZPC3, and ZPC4 microfibers. All insets are shown at 5000× of magnification. (b) Analysis of the diameter of the fibers. (c) ATR-FTIR spectra of the ZPC composite microfibers and the single components (zein, pectin, soy lecithin, and VitC). The peaks highlighted in the red area on ZPCs correspond to the protein component, while the bands in the yellow areas indicate the soy lecithin molecule presence inside the samples. (d) Chemical structures of zein, pectin, soy lecithin, and VitC. (e) Diameter analysis of the non-cross-linked and the CaCl_2_-cross-linked ZPC0 fibers. Asterisks represent statistical significance (**p < 0.01 and ***p < 0.001). CL = cross-linking. (f) SEM images of non-cross-linked and CaCl_2_-cross-linked ZPC0 fibers, acquired after the treatment.

The morphology of the prepared fibrous scaffolds was investigated under SEM. The fibrous samples look homogeneous, with smooth fibers covering the entire surface of the target and without the presence of beads, as shown in Figure 1a. Moreover, the microfibers look continuous and smooth with a ribbon-like structure. This structure was already noticed in other studies, in which zein was used at the same concentration as the present work.^46,47^ Ribbon-like fibers were produced as a consequence of the high volatility of the solvent used in the process (ethanol), which leads to the formation of an outer tube that then collapses following the evaporation of the residual solvent, thus resulting in a final flat structure.^48^ Furthermore, SEM inspection demonstrated that the VitC did not affect the morphology of the fibers.

Average dimensions, determined using ImageJ software and analyzing ca. 100 fibers per image, resulted to be 0.84 ± 0.03, 0.91 ± 0.04, 0.98 ± 0.03, 1.07 ± 0.04, and 1.19 ± 0.06 μm for ZPC0, ZPC1, ZPC2, ZPC3, and ZPC4, respectively (Figure 1b). These data suggested that the diameter of the fibers was proportional to the concentrations of VitC loaded inside the samples. Our fabricated microfibers resulted slightly bigger compared to the average diameter of the fibers constituting the natural ECM, which ranges from 12 nm to more than 500 nm, depending on the stage of development.^49^ Nevertheless, Erisken and co-workers demonstrated that microfibers can promote cell alignment and even boost the overall deposition of the various ECM components.^50^

ATR-FTIR was used to characterize the intramolecular interactions within the samples. The FTIR spectra of the ZPC microfibers (Figure 1c) preserved some of the peaks corresponding to the presence of zein (Figure 1c, dashed brown line): 2800 and 3500 cm^–1^ of the amide A; 1639 cm^–1^ of the amide I; and 1532 cm^–1^ of the amide II corresponding to the N–H bond of the protein aromatic ring.^51^ The chemical formula of the molecules used is reported in Figure 1d. Some bands indicated the presence of the emulsifying agent (Figure 1c, dashed yellow line) in the final product, as noticeable from the 2920 and 2854 cm^–1^ signals of the symmetric and antisymmetric CH_2_ bend and by the C=O stretching vibration at 1730 cm^–1^. In addition, the PO^2–^ and P–O–C infrared active vibrations of the soy lecithin at 1050 cm^–1^ are identified in the samples, confirming the presence of the emulsion agent into the microfibers.^52^ It is important to mention that the FTIR characterization did not allow the identification of the characteristic peaks pertaining to either pectin or ascorbic acid (Figure 1c, dashed light-blue and dashed purple line, respectively) as they overlapped with the signals arising from zein (the predominant component) and soy lecithin. Finally, no shifting of the bands occurred, suggesting that no chemical bonds took place between the components during the electrospinning process.

Assuming that the pectin would be homogeneously distributed in the produced microfibers (also thanks to the addition of soy lecithin included in the emulsion), the ability of this polysaccharide to cross-link was investigated through various approaches, in order to increase the stability of the samples in an aqueous environment. The exposition to divalent metal ions such as Ca^2+^ leads to the CL of the pectin molecules through the “egg-box” model.^29,53,54^ In another study, it was observed how TFA promotes the protonation of the carboxyl group of the polysaccharide, reducing its solubility in water.^55^ For these reasons, three different approaches were tested, consisting of (a) immersion in a CaCl_2_ solution, exposition to TFA-saturated environment (b) without, and (c) with subsequent washing steps. The analysis of the treated fibers morphology and dimensions, together with a preliminary cytocompatibility screening highlighted the superior efficacy of the CL with CaCl_2_ (Figure S2a,c). Even though the CL with TFA appeared to lead to optimal structure maintenance (Figure S2b), the system was characterized by a significant decrease in cell viability (%), compared to the control (Figure S2c). On the contrary, the CL with TFA followed by two washing steps in PBS presented good biocompatibility (Figure S2c) but a loss of 3D structure (Figure S2b). For these reasons, all the following analyses were performed onto ZPC microfibers cross-linked with CaCl_2_ (defined as ZPC_CL_ in the text), whose morphological features are reported in detail in Figure 1e,f.

### 3.2 Vitamin C Release Profile and Anti-oxidant Activity

**Figure 2:**
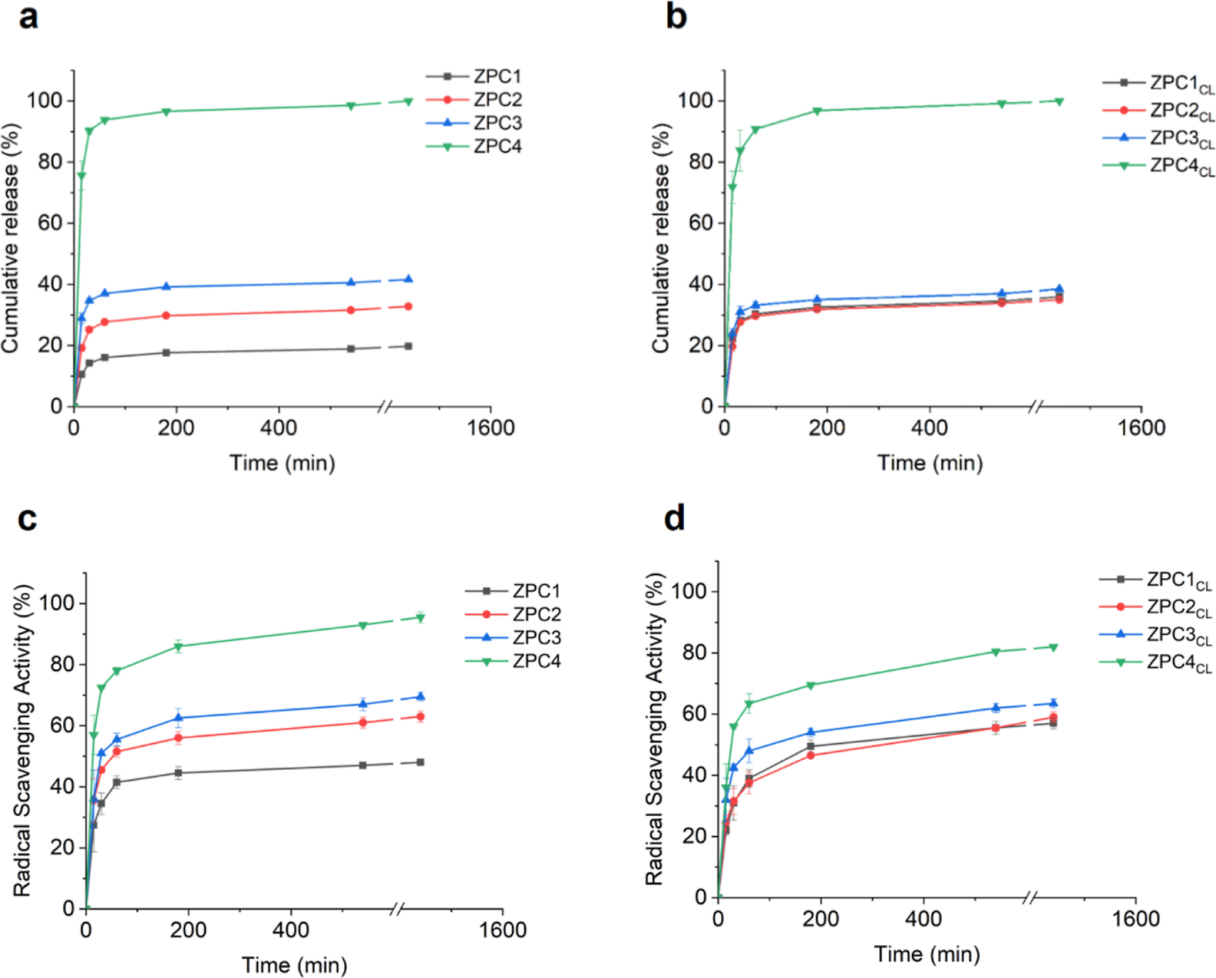
Vitamin C identification. Cumulative release (%) of VitC form (a) non-cross-linked and (b) CaCl_2_-cross-linked microfibers after 24 h. Radical scavenging activity (%) against DPPH^•^ free radical of VitC-loaded (c) non-cross-linked and (d) CaCl_2_-cross-linked ZPC microfibers.

The cumulative release profile of VitC from the non-cross-linked and cross-linked microfibers is depicted in Figure 2a,b. As expected, the release after 24 h of immersion in PBS at 37 °C was proportional to the amount of VitC loaded inside the samples and thus resulted significantly higher for ZPC4/ZPC4_CL_ microfibers compared to microfibers with lower concentrations of encapsulated VitC, both non-cross-linked and cross-linked. In all samples, a burst release in the buffer occurred within 3 h of immersion. This behavior was ascribable to the easy diffusion of the VitC from the scaffolds to the external aqueous environment.^56^

The antioxidant activity of the VitC is well documented^38,57^ and was herein monitored with the DPPH^**•**^ free-radical scavenging assay. As shown in Figure 2c,d, the free-radical scavenging activity was visible for all the tested samples, and the percentage evaluated after 24 h was proportional to the amount of VitC loaded into the microfibrous matrices (ZPC4_CL_ > ZPC3_CL_ > ZPC2_CL_ > ZPC1_CL_), as expected. Moreover, the pattern of the curves of the samples looked consistent with the one obtained from the release assay of VitC (Figure 2a,b), providing further important information about the amount of the bioactive molecule released from the samples. Indeed, the VitC released in the external environment was immediately available to exert its antioxidant activity.

### 3.3 Degradation and Swelling Analysis of the Composite Microfibers

**Figure 3:**
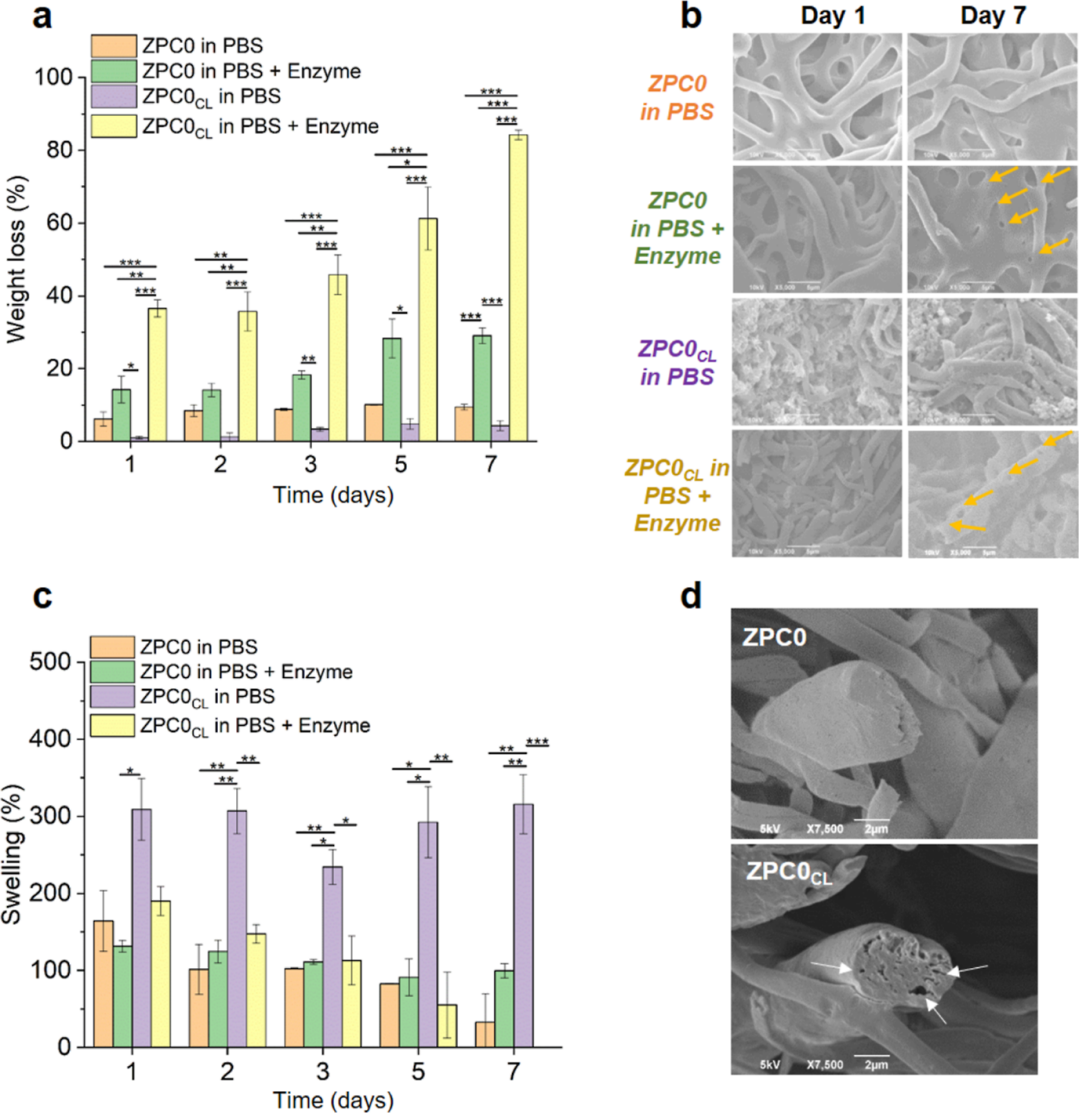
Water response of the composite fibers. (a) Weight loss (%) of the ZPC0 and ZPC0_CL_ microfibers up to 7 days of incubation in PBS alone or in PBS supplemented with Protease XIV from *Streptomyces griseus* (≥3.5 units/mg). (b) SEM images of ZPC0 and ZPC0_CL_ microfibers after 1 and 7 days of incubation under the conditions previously described. Yellow arrows indicate the Protease activity on the protein component of the sample (visible as nanopores). (c) Swelling capacity (%) of the ZPC0 and ZPC0_CL_ microfibers up to 7 days of incubation in PBS alone or in PBS supplemented with Protease XIV from *Streptomyces griseus*. On day 7, it was not possible to record the swelling of the ZPC0_CL_ sample in PBS + Enzyme due to the enzymatic effect. (d) SEM images of the ZPC0 and ZPC0_CL_ cross-sections. White arrows indicate the holes generated after pectin cross-linking. Asterisks represent statistical significance (*p < 0.05, **p < 0.01, and ***p < 0.001).

Each component of our designed composite microfibers is biodegradable. Zein, which normally does not degrade in a short time,^58^ undergoes a degradation process when exposed to an enzymatic action.^59^ Since the wound bed contains abundant levels of proteases, essential for proper tissue regeneration,^60^ it was of interest to analyze the fiber degradation behavior in the presence of such enzymatic environment mimicking the in vivo condition as closely as possible. Experiments were performed onto ZPC0/ZPC0_CL_ samples immersed in PBS at 37 °C, either in the presence or absence of Protease XIV from *Streptomyces*, a proteolytic enzyme able to hydrolyze peptide bonds on the carboxyl side of glutamic or aspartic acid.^61^ The degradation of the protein scaffold (Figure 3a) resulted significantly higher (*p < 0.05, **p < 0.01, and ***p < 0.001) for both the non-cross-linked and the cross-linked microfibers when the samples were immersed in PBS supplemented with the protease, as expected. However, such difference was much more pronounced for ZPC0_CL_ samples, which reached a limited weight loss (∼10%) after 7 days of immersion in PBS alone (Figure 3a, purple columns), while almost 90% of the ZPC0_CL_ sample weight was lost when exposed for the same time to the enzymatic activity (Figure 3a, yellow columns). The effect of the Protease XIV from *S. griseus* on the samples was investigated also by SEM imaging, as seen in Figure 3b. The micrographs highlighted a loss of the normal scaffold architecture on ZPC0 and more on ZPC0_CL_ after 7 days of immersion in PBS with the protease, compared to the same samples immersed in the buffer alone. The microfibrous mats tended to become film-like and disappear over time, a process already described in the literature.^62^

Swelling analysis was carried out in parallel with the degradation test under the same conditions described above. The results, presented in Figure 3c, highlighted the general tendency of all the samples to reduce the PBS uptake over time, except for the cross-linked ones when immersed in PBS alone (Figure 3c, purple columns). Indeed, under this condition, ZPC0_CL_ showed a very high percentage of swelling until 1 week of immersion in the buffer. These data suggested that the microfibers may acquire a hydrogel behavior when cross-linked, being able to soak up the buffer and swell, increasing their weight up to ∼300% from day 1 to day 7. To better understand this aspect, the cross-sections of ZPC0 and ZPC0_CL_ samples were examined under the scanning electron microscope (Figure 3d). The microscopic investigation revealed the presence of holes within the structure of a single, cross-linked microfiber (Figure 3d, white arrows), which were absent in the non-cross-linked ones. These results confirmed that the pectin contained inside the scaffolds was cross-linked and, most importantly, that the molecular network obtained was able to uptake PBS as efficiently as a hydrogel. At the same time, the CL modification led to a faster fibers’ degradation when exposed to the protease (Figure 3c, yellow columns). A possible hypothesis to explain these (apparently conflicting) behaviors is that the enzyme may exert more efficiently its activity on the ZPC0_CL_ samples by infiltrating into the holes and open spaces within the cross-linked structure, thus coming in contact with a larger surface area. This behavior could be associated to two different contributions: (1) the immersion in a water-like medium facilitates the swelling of the gelled polysaccharide; (2) the formation of hydrogel areas within the structure of the microfibers leads to higher absorption of the aqueous solution, promoting the proteolytic activity toward the protein component (zein).

### 3.4 In Vitro Biocompatibility and Cell Morphology Investigation

**Figure 4:**
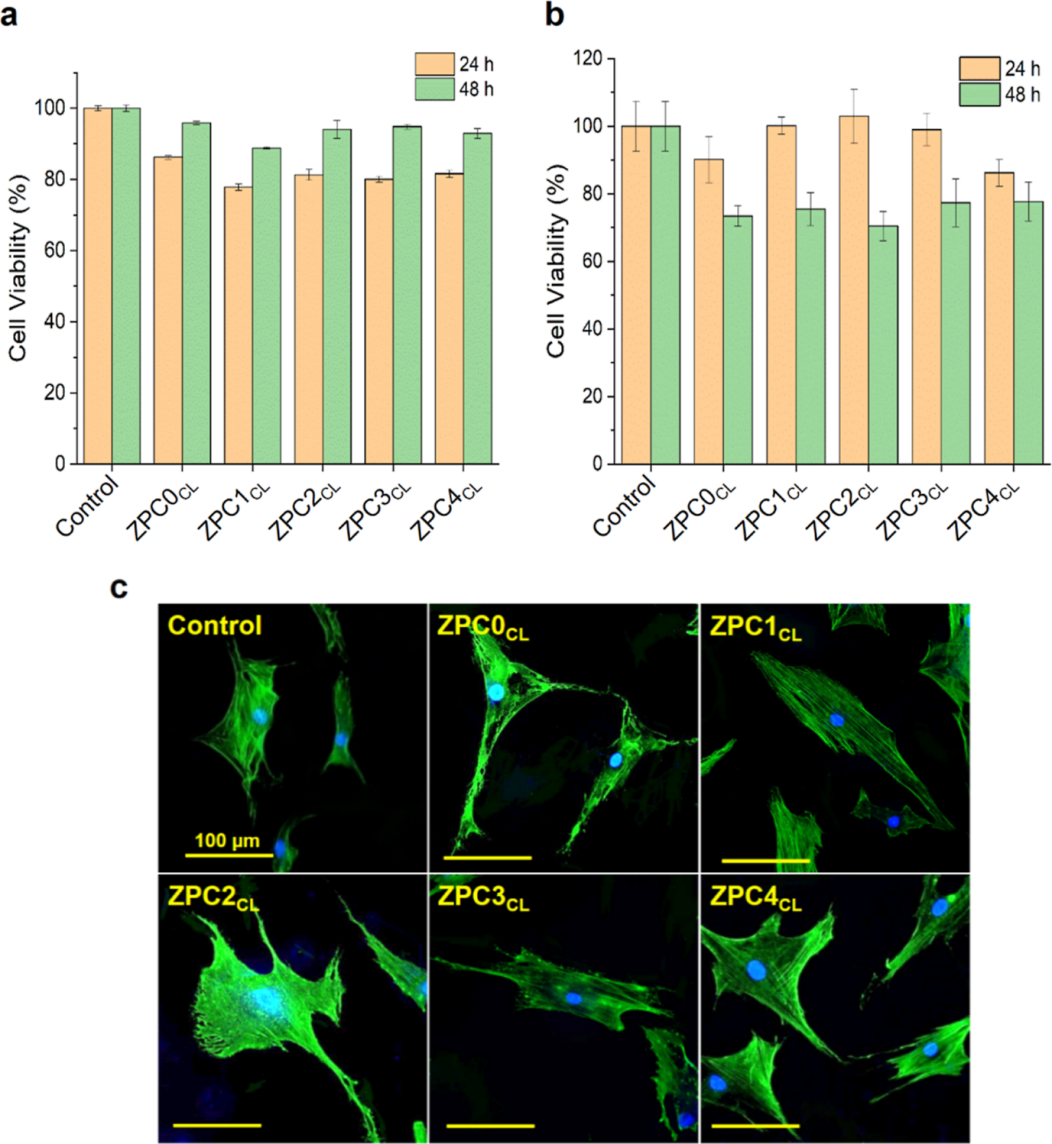
In vitro biocompatibility of the composite fibers extracts from CL microfibers. Cell viability assay (a) on HDFa cells and (b) on keratinocytes (HaCaT) cells after 24 and 48 h of growth in the presence of the extracts obtained from the CL samples. (c) Confocal images depicting the HDFa cell morphology after 48 h of growth in the presence of the extraction media of the different ZPCs_CL_ samples. Scale bar of 100 μm.

The biocompatibility of the ZPCs_CL_ samples (Figure 4a,b) was performed in vitro on HDFa cells and on primary human keratinocytes (HaCaT) cells. HDFa presented a slight decrease in cell viability compared to the control, when grown for 24 h in the presence of the extraction media obtained from the cross-linked microfibers (Figure 4a, orange columns). Nevertheless, the number of viable cells was fully recovered after 48 h of growth in the same condition (Figure 4a, green columns). HaCaT cells showed a decrease in biocompatibility over time compared to the control, reaching about 80% of cell viability after 48 h of growth in the extraction medium (Figure 4b). Overall, the data revealed an optimal cytocompatibility for both cell populations. Similar results were also obtained for the case of the extraction media from the non-cross-linked microfibers, as reported in Figure S3 (Supporting Information).

HDFa morphology was investigated with confocal microscopy, to analyze the state of health of the cells after 48 h of growth in the presence of the different extraction media. The images are depicted in Figure 4c and show numerous healthy cells. Indeed, fibroblasts presented the typical elongated shape, further validating the in vitro biocompatibility outcomes.

### 3.5 In Vitro Antioxidant Assay onto Human Keratinocytes

**Figure 5:**
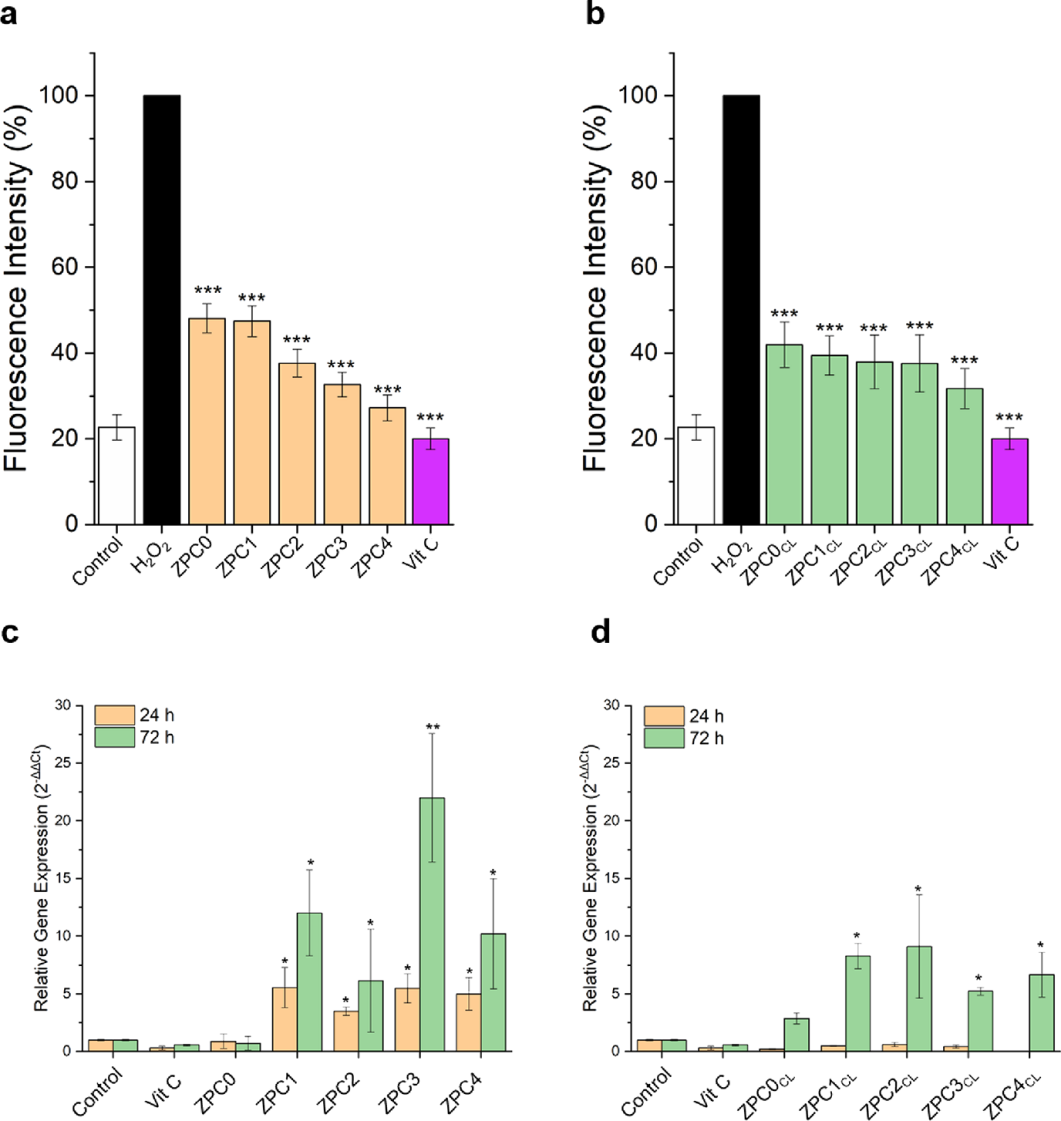
In vitro bioactivity tests. In vitro antioxidant DCFH-DA assay onto HaCaT cells after 24 h of growth in the presence of the extraction media obtained from (a) non-CL and (b) CL microfibers. The black column corresponds to the H_2_O_2_ positive control. Collagen 1a (*col*-*1a*) gene expression obtained via Rt-qPCR on the mRNA extracted from HDFa cells after 1 and 3 days of growth in the presence of the extraction medium obtained from (c) non-CL and (d) CL microfibers. Asterisks represent statistical significance with respect to the control samples (*p < 0.05, **p < 0.005, and ***p < 0.001).

The antioxidant activity of the VitC released from the cross-linked and non-cross-linked microfibers was also analyzed in vitro on HaCaT cells through the DCFH-DA assay fluorescent probe, an oxidation-sensitive test used as a general marker of intracellular ROS.^63^ The output of the DCFH-DA assay is reported in Figure 5a. The results highlighted a decrease in the fluorescence intensity directly proportional to the amount of VitC loaded inside the fibrous mats (down to 50 and 30%, for the ZPC0 and the ZPC4 medium extracts, respectively). Such fluorescence decline corresponds to higher antioxidant activity toward intracellular ROS, confirming the ability of the released VitC to protect the keratinocytes from the oxidative stress. This trend is much more marked for the non-cross-linked microfibers (Figure 5a) compared to the cross-linked ones (Figure 5b), as already seen for the DPPH^**•**^ antioxidant assay previously described. These outcomes may be connected to the CL steps performed by immersion of the samples in CaCl_2_ solution, which could facilitate a pre-release of a small amount of VitC, resulting in a decrease of its antioxidant activity of HaCaT cells.

### 3.6 Real-Time qPCR Analysis for ECM Protein Gene Expression

The ability of the VitC released from the samples to stimulate the gene expression of the collagen protein was investigated.^42^ Real-time PCR analyses were performed on the mRNA extracted from the HDFa cells grown in the presence of the extraction media obtained from the cross-linked and non-cross-linked microfibers. The relative gene expression (2^–ΔΔ*Ct*^) was assessed after 24 and 72 h, and the final outputs are exhibited in Figure 5c,d, for cross-linked and non-cross-linked fibers, respectively. Both cross-linked and non-cross-linked samples showed an increase in *col-1a* expression when VitC was present in the microfibers. However, the amount of VitC appeared not directly proportional to the level of gene expression. Furthermore, lower values of *col-1a* gene expression were recorded for cells grown for 24 h in the presence of the extraction media obtained from the cross-linked samples compared to the culture treated with the extraction media of the non-cross-linked samples. Most likely, this outcome is due to the slower release of VitC from the cross-linked microfibers, as a consequence of the CaCl_2_ CL, which should allow the prolongation of the ECM synthesis in vivo and enhance the tissue repairing. For each studied condition, a statistically significant increase of col-1a gene expression occurred after 3 days of cell growth in the extraction media compared to the control, indicating that the VitC released from the microfibers was able to stimulate the collagen mRNA production overtime. Moreover, the stimulation of collagen seems to be dependent exclusively on the VitC since the expression of *col-1a* was null for ZPC0/ZPC0_CL_ compared to the other samples bearing the bioactive molecule. This result is even more significant considering that pectin and soy lecithin (the two water-soluble microfibers components) were observed to be involved in a decrease of collagen mRNA levels in normal human dermal fibroblast cells and skeletal muscle cells^64,65^ Furthermore, ZPC3 and ZPC3_CL_ have shown a significant 25-fold and 5-fold increase, respectively, in *col-1* relative gene expression compared to the control (Figure 5c,d).

In addition, the relative gene expression of *bcl-2* and *bax* were monitored on cells kept in contact with cross-linked and non-cross-linked microfiber extracts for 24 and 72 h. Both genes are namely involved in cellular live/death processes. While *bcl-2* enhances cell survival by suppressing apoptosis, *bax* expression is essential for the activation of apoptosis in normal cells.^66,67^ The results revealed *bcl-2* and *bax* expression levels lower in substrates treated for 72 h than those treated with the extractants for 24 h. Moreover, the *bcl-2* expression was higher for the non-cross-linked samples, compared to the cross-linked ones (Figure S4a,b, Supporting Information), suggesting a stronger anti-apoptotic activity of *bcl-2* gene in the absence of the CL procedure. Furthermore, an increasing trend of *bcl-2* expression, proportional to the amount of the VitC content, was recorded for the non-cross-linked samples. In particular, ZPC3 seemed to hinder the cellular apoptosis and showed a gene overexpression comparable to cells treated with VitC alone. Even if a lower level of *bcl-2* expression occurred with cells treated with the cross-linked microfibers, the expression levels did not differ significantly from the control, suggesting that the extracts did not impair the normal cell homeostasis. No *bax* overexpression was registered in both cells treated with the cross-linked samples (Figure S4d, Supporting Information) and non-cross-linked samples, showing approximately the same values (Figure S4c, Supporting Information). This indicates that no pro apoptotic effects occurred on cells treated with both cross-linked and non-cross-linked VitC-loaded microfibers. The significant increase of *bax* expression recorded only in ZPC3 and ZPC4 compared to the control at 24 h is defeated by the concomitant expression of *bcl-2*, demonstrating a balanced ratio in apoptosis regulation.

Overall, these data suggested that ZPC3 and ZPC3_CL_ microfibers were promising for the next in vitro and in vivo analyses.

### 3.7 In Vitro Cell Attachment and Vivo Study on UVB-Induced Skin Inflammation

**Figure 6:**
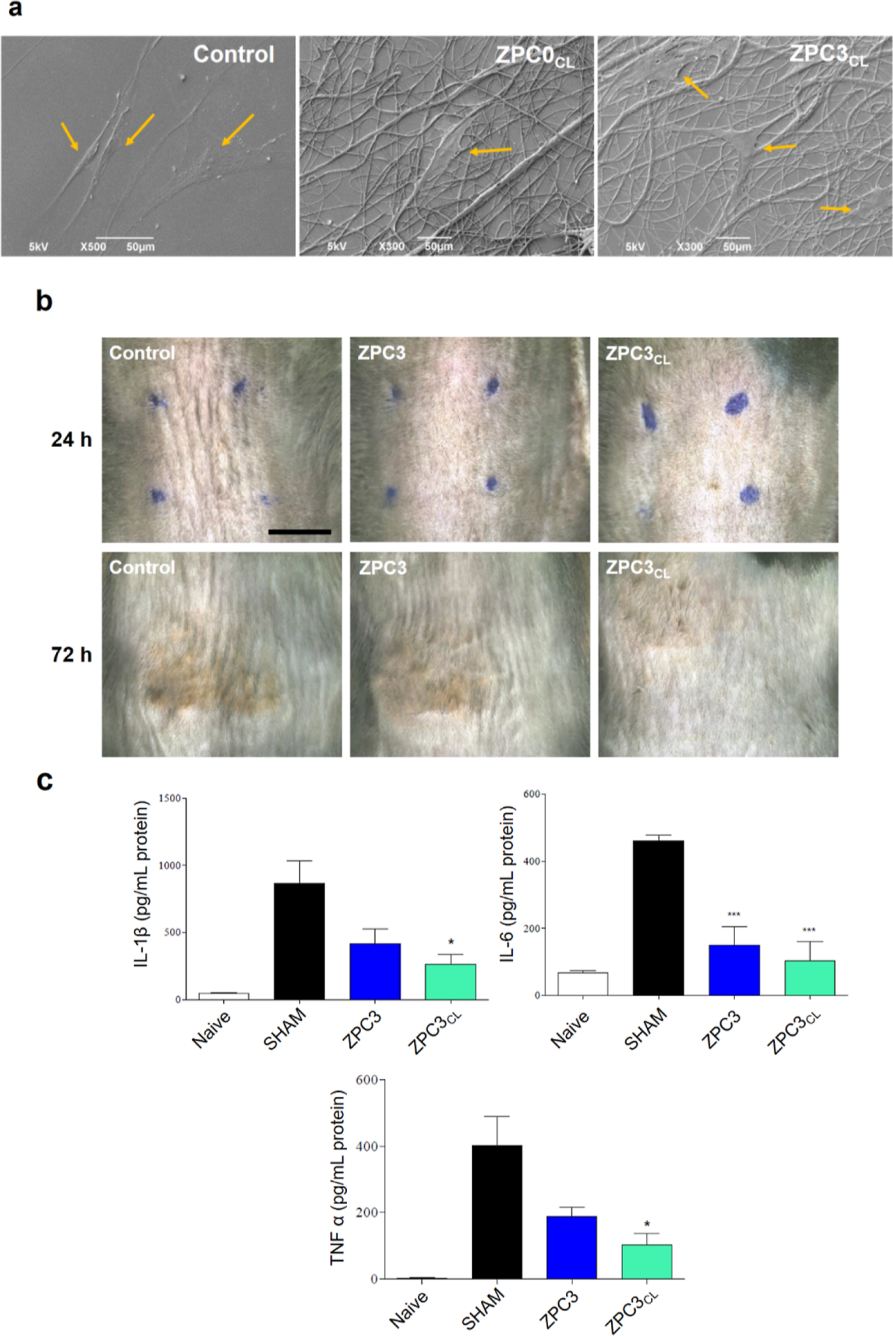
(a) Direct plating of the HDFa cells onto glass coverslips (control), ZPC0_CL_, and ZPC3_CL_ microfibers. Orange arrows indicated the HDFa cells. (b) Photographs of the mice UVB-burned skin after 24 and 72 h of treatment with ZPC3 and ZPC3_CL_ microfibers. The blue dots show the application area of the plant-based patches on the mice skin. Scale bar of 0.5 mm. (c) ELISA measurements for the quantification of the cytokines IL-1β, IL-6, and TNF-α expression in the mice skin burned area. Asterisks represent statistical significance with respect to the control, SHAM samples (*p < 0.05 and ***p < 0.001).

Based on the above presented results, ZPC0_CL_ and ZPC3_CL_ were selected to test their ability to serve as cell adhesion artificial matrices and anti-inflammatory skin patches. Besides being biocompatible, a wound dressing must also promote cell attachment, growth, and proliferation. The adhesion of HDFa cells on the cross-linked microfibers ZPC0_CL_ and ZPC3_CL_ after direct culture for 48 h was investigated by SEM (Figure 6a). HDFa cells remained well attached to the cross-linked microfibrous scaffolds, indicating a good interaction among the material and the cells.

A mouse model of mild, UVB-skin burn was used to evaluate the anti-inflammatory properties of the VitC-loaded patches and to ultimately validate the application of our developed materials. As shown in Figure 6b, after UVB exposure, mice skin developed redness and erythema. This cutaneous manifestation was also associated with an increase in the production of pro-inflammatory cytokines (Figure 6c). Vit-C-loaded microfibers, ZPC3 and ZPC3_CL_, were applied over the irradiated mouse skin immediately after exposure to the irritating light, and cutaneous levels of pro-inflammatory cytokines (IL-6, IL-1β, and TNF-α) were assessed at 48 h post irradiation and compared to the ones obtained for a naïve sample (mouse not exposed to the UVB light). The application of the hydrogel-like, plant-based non cross-linked patch significantly reduced the expression of all cytokines as compared to the untreated group. Specifically, the expressions of IL-6, IL-1β, and TNF-α were up to 50% lower than that for the untreated group. An even stronger reduction was also observed for the animals treated with ZPC3_CL_ microfiber patches.

## 4 Conclusions

In this work, plant-based microfibrous scaffolds were successfully fabricated via electrospinning of various emulsion blends of zein, pectin, and multiple concentrations of VitC. Samples were also subjected to a CL process, exploiting the pectin ability to gel under specific conditions. With the optimization of the post-fabrication treatment, CL in the presence of Ca^2+^ cations resulted in the best outcome in terms of morphology, diameter sizes, and biocompatibility. All the electrospun scaffolds presented smooth surfaces and bead-free morphology, with the average diameter directly proportional to the loaded VitC.

FTIR analysis confirmed the presence of zein as the main component of the samples, but it was not able to highlight the peaks corresponding to the VitC. However, this bioactive molecule was identified by monitoring its release from the microfibers and its antioxidant functionality. As expected, the release and the radical scavenging activity were more remarkable for the samples bearing higher concentrations of the bioactive molecule.

Degradation and swelling ability were evaluated after immersion in PBS alone or PBS supplemented with a protease. In general, the presence of the enzyme in the buffer significantly increased the degradation rate of the samples, which was considerably higher for the cross-linked scaffolds. Indeed, the swelling profile tended to zero for the cross-linked microfibers due to their complete degradation in the presence of the protease. On the contrary, the cross-linked microfibers immersed in PBS alone acted as hydrogel due to its ability to absorb a very high volume of the buffer. Cross-sections of the samples were performed by SEM, demonstrating that the post-fabrication treatment led to hole formation in the microfiber internal structure. A possible hypothesis is that the pectin domains dispersed in the cross-linked samples behave as “hydrogel-like islands” within the polymeric matrix. When immersed in the buffer, these islands swell considerably and constitute homogeneously dispersed areas which are more susceptible to the protease activity. Eventually, this leads to a faster degradation rate compared to the non-cross-linked samples.

The in vitro biocompatibility was confirmed on HDFa and HaCaT cells through metabolic assays and confocal imaging. Furthermore, direct plating of the fibroblasts onto the microfibers demonstrated the ability of these cells to attach to the scaffolds, maintaining their typical elongated shape. In vitro DCFH-DA assay was performed to evaluate the antioxidant property of the released VitC on the HaCat cells. The data highlighted a common trend between the cross-linked and non-cross-linked samples in reducing the oxidative stress proportionally to the concentration of VitC present in the scaffolds and released from the microfibers. Furthermore, the bioactive molecule selected for this study was able to stimulate the synthesis of collagen promoting its gene expression, as well as modulate the expression of *bcl-2* and *bax* genes to hinder the apoptotic process in the fibroblast cells, as demonstrated by the real-time PCR analysis.

Lastly, in vivo tests on a mice model of UVB-induced skin burn were performed. Visual analysis of the skin and the ELISA quantification of the inflammatory cytokines, involved in the burn healing process, strongly confirmed the ability of the tested samples to promote the healing process and reduce the inflammation in the damaged skin. Overall, the results of the presented investigation revealed that our designed and developed, plant-based, hydrogel-like microfibers constitute a suitable, naturally derived alternative, as anti-oxidant and anti-inflammatory platform for skin wound care.

## Supporting Information Available

Composite fiber fabrication workflow from the solution preparation to the electrospinning process; CL strategy optimization, with assessment of the microfibers morphology and their cell compatibility; in vitro biocompatibility of the non-cross-linked microfiber extracts with fibroblasts and keratinocytes, and real-time PCR analysis of *bcl-2* and *bax* genes (PDF)

## References

[ref1] Enoch S.; Leaper D. J. Basic Science of Wound Healing. Surgery 2008, 26, 31–37. 10.1016/j.mpsur.2007.11.005.

[ref2] Rodriguez P. G.; Felix F. N.; Woodley D. T.; Shim E. K. The Role of Oxygen in Wound Healing: A Review of the Literature. Dermatol. Surg. 2008, 34, 1159–1169. 10.1111/j.1524-4725.2008.34254.x. 18513296

[ref3] Schäfer M.; Werner S. Oxidative Stress in Normal and Impaired Wound Repair. Pharmacol. Res. 2008, 58, 165–171. 10.1016/j.phrs.2008.06.004. 18617006

[ref4] Dunnill C.; Patton T.; Brennan J.; Barrett J.; Dryden M.; Cooke J.; Leaper D.; Georgopoulos N. T. Reactive Oxygen Species (ROS) and Wound Healing: The Functional Role of ROS and Emerging ROS-Modulating Technologies for Augmentation of the Healing Process. Int. Wound J. 2017, 14, 89–96. 10.1111/iwj.12557. 26688157 PMC7950185

[ref5] Maquart F. X.; Monboisse J. C. Extracellular Matrix and Wound Healing. Pathol. Biol. 2014, 62, 91–95. 10.1016/j.patbio.2014.02.007. 24650524

[ref6] Boot-Handford R. P.; Tuckwell D. S. Fibrillar Collagen: The Key to Vertebrate Evolution? A Tale of Molecular Incest. BioEssays 2003, 25, 142–151. 10.1002/bies.10230. 12539240

[ref7] Xue M.; Jackson C. J. Extracellular Matrix Reorganization During Wound Healing and Its Impact on Abnormal Scarring. Adv. Wound Care 2015, 4, 119–136. 10.1089/wound.2013.0485. PMC4352699 25785236

[ref8] Rousselle P.; Montmasson M.; Garnier C. Extracellular Matrix Contribution to Skin Wound Re-Epithelialization. Matrix Biol. 2019, 75–76, 12–26. 10.1016/j.matbio.2018.01.002. 29330022

[ref9] Thangavel P.; Vilvanathan S. P.; Kuttalam I.; Lonchin S. Topical Administration of Pullulan Gel Accelerates Skin Tissue Regeneration by Enhancing Collagen Synthesis and Wound Contraction in Rats. Int. J. Biol. Macromol. 2020, 149, 395–403. 10.1016/j.ijbiomac.2020.01.187. 31978478

[ref10] Lei H.; Fan D. Conductive, Adaptive, Multifunctional Hydrogel Combined with Electrical Stimulation for Deep Wound Repair. Chem. Eng. J. 2021, 421, 129578 10.1016/j.cej.2021.129578.

[ref11] Ambekar R. S.; Kandasubramanian B. Advancements in Nanofibers for Wound Dressing: A Review. Eur. Polym. J. 2019, 117, 304–336. 10.1016/j.eurpolymj.2019.05.020.

[ref12] Wang Z.; Crandall C.; Sahadevan R.; Menkhaus T. J.; Fong H. Microfiltration Performance of Electrospun Nanofiber Membranes with Varied Fiber Diameters and Different Membrane Porosities and Thicknesses. Polymer 2017, 114, 64–72. 10.1016/j.polymer.2017.02.084.

[ref13] Ding J.; Zhang J.; Li J.; Li D.; Xiao C.; Xiao H.; Yang H.; Zhuang X.; Chen X. Electrospun Polymer Biomaterials. Prog. Polym. Sci. 2019, 90, 1–34. 10.1016/j.progpolymsci.2019.01.002.

[ref14] Suarato G.; Bertorelli R.; Athanassiou A. Borrowing From Nature: Biopolymers and Biocomposites as Smart Wound Care Materials. Front. Bioeng. Biotechnol. 2018, 6, 1–11. 10.3389/fbioe.2018.00137. 30333972 PMC6176001

[ref15] Bilirgen A. C.; Toker M.; Odabas S.; Yetisen A. K.; Garipcan B.; Tasoglu S. Plant-Based Scaffolds in Tissue Engineering. ACS Biomater. Sci. Eng. 2021, 7, 926–938. 10.1021/acsbiomaterials.0c01527. 33591719

[ref16] Zamri M. F. M. A.; Bahru R.; Amin R.; Aslam Khan M. U.; Razak S. I. A.; Hassan S. A.; Kadir M. R. A.; Nayan N. H. M. Waste to Health: A Review of Waste Derived Materials for Tissue Engineering. J. Clean. Prod. 2021, 290, 125792 10.1016/j.jclepro.2021.125792.

[ref17] Suarato G.; Contardi M.; Perotto G.; Heredia-Guerrero J. A.; Fiorentini F.; Ceseracciu L.; Pignatelli C.; Debellis D.; Bertorelli R.; Athanassiou A. From Fabric to Tissue: Recovered Wool Keratin/Polyvinylpyrrolidone Biocomposite Fibers as Artificial Scaffold Platform. Mater. Sci. Eng., C 2020, 116, 111151 10.1016/j.msec.2020.111151. 32806258

[ref18] Trojanowska D. J.; Suarato G.; Braccia C.; Armirotti A.; Fiorentini F.; Athanassiou A.; Perotto G. Wool Keratin Nanoparticle-Based Micropatterns for Cellular Guidance Applications. ACS Appl. Nano Mater. 2022, 5, 15272–15287. 10.1021/acsanm.2c03116. 36338329 PMC9624257

[ref19] Chandika P.; Ko S. C.; Jung W. K. Marine-Derived Biological Macromolecule-Based Biomaterials for Wound Healing and Skin Tissue Regeneration. Int. J. Biol. Macromol. 2015, 77, 24–35. 10.1016/j.ijbiomac.2015.02.050. 25783018

[ref20] Martău G. A.; Mihai M.; Vodnar D. C. The Use of Chitosan, Alginate, and Pectin in the Biomedical and Food Sector—Biocompatibility, Bioadhesiveness, and Biodegradability. Polymers 2019, 11, 1837 10.3390/polym11111837. 31717269 PMC6918388

[ref21] Souza M. A. D.; Vilas-Boas I. T.; Leite-da-Silva J. M.; Abrahão P. d. N.; Teixeira-Costa B. E.; Veiga-Junior V. F. Polysaccharides in Agro-Industrial Biomass Residues. Polysaccharides 2022, 3, 95–120. 10.3390/polysaccharides3010005.

[ref22] Jaski A. C.; Schmitz F.; Horta R. P.; Cadorin L.; da Silva B. J. G.; Andreaus J.; Paes M. C. D.; Riegel-Vidotti I. C.; Zimmermann L. M. Zein - a Plant-Based Material of Growing Importance: New Perspectives for Innovative Uses. Ind. Crops Prod. 2022, 186, 115250 10.1016/j.indcrop.2022.115250.

[ref23] Naqash F.; Masoodi F.; Rather S. A.; Wani S.; Gani A. Emerging Concepts in the Nutraceutical and Functional Properties of Pectin – A Review. Carbohydr. Polym. 2017, 168, 227–239. 10.1016/j.carbpol.2017.03.058. 28457445

[ref24] Cui J.; Qiu L.; Qiu Y.; Wang Q.; Wei Q. Co-Electrospun Nanofibers of PVA-SbQ and Zein for Wound Healing. J. Appl. Polym. Sci. 2015, 132, 1–9. 10.1002/app.42565.25866416

[ref25] Kimna C.; Tamburaci S.; Tihminlioglu F. Novel Zein-Based Multilayer Wound Dressing Membranes with Controlled Release of Gentamicin. J. Biomed. Mater. Res., Part B 2018, 107, 2057–2070. 10.1002/jbm.b.34298. 30576095

[ref26] Dashdorj U.; Reyes M. K.; Unnithan A. R.; Tiwari A. P.; Tumurbaatar B.; Park C. H.; Kim C. S. Fabrication and Characterization of Electrospun Zein/Ag Nanocomposite Mats for Wound Dressing Applications. Int. J. Biol. Macromol. 2015, 80, 1–7. 10.1016/j.ijbiomac.2015.06.026. 26093320

[ref27] Babitha S.; Korrapati P. S. Biodegradable Zein–Polydopamine Polymeric Scaffold Impregnated with TiO 2 Nanoparticles for Skin Tissue Engineering. Biomed. Mater. 2017, 12, 055008 10.1088/1748-605X/aa7d5a. 28944761

[ref28] Masmoudi M.; Besbes S.; Abbes F.; Robert C.; Paquot M.; Blecker C.; Attia H. Pectin Extraction from Lemon By-Product with Acidified Date Juice: Effect of Extraction Conditions on Chemical Composition of Pectins. Food Bioprocess Technol. 2012, 5, 687–695. 10.1007/s11947-010-0344-2.

[ref29] Cao L.; Lu W.; Mata A.; Nishinari K.; Fang Y. Egg-Box Model-Based Gelation of Alginate and Pectin: A Review. Carbohydr. Polym. 2020, 242, 116389 10.1016/j.carbpol.2020.116389. 32564839

[ref30] Lin H.; Chen H.-H.; Chang S.-H.; Ni T.-S. Pectin-Chitosan-PVA Nanofibrous Scaffold Made by Electrospinning and Its Potential Use as a Skin Tissue Scaffold. J. Biomater. Sci. Polym. Ed. 2013, 24, 470–484. 10.1080/09205063.2012.693047. 23565688

[ref31] Fiorentini F.; Suarato G.; Grisoli P.; Zych A.; Bertorelli R.; Athanassiou A. Plant-Based Biocomposite Films as Potential Antibacterial Patches for Skin Wound Healing. Eur. Polym. J. 2021, 150, 110414 10.1016/j.eurpolymj.2021.110414.

[ref32] Sadeghi M. Pectin-Based Biodegradable Hydrogels with Potential Biomedical Applications as Drug Delivery Systems. J. Biomater. Nanobiotechnol. 2011, 02, 36–40. 10.4236/jbnb.2011.21005. 11996036

[ref33] Giusto G.; Vercelli C.; Comino F.; Caramello V.; Tursi M.; Gandini M. A New , Easy-to-Make Pectin-Honey Hydrogel Enhances Wound Healing in Rats. BMC Complementary Altern. Med. 2017, 17, 266 10.1186/s12906-017-1769-1. PMC5433168 28511700

[ref34] Pereira R. F.; Barrias C. C.; Bártolo P. J.; Granja P. L. Cell-Instructive Pectin Hydrogels Crosslinked via Thiol-Norbornene Photo-Click Chemistry for Skin Tissue Engineering. Acta Biomater. 2018, 66, 282–293. 10.1016/j.actbio.2017.11.016. 29128530

[ref35] Munarin F.; Tanzi M. C.; Petrini P. Advances in Biomedical Applications of Pectin Gels. Int. J. Biol. Macromol. 2012, 51, 681–689. 10.1016/j.ijbiomac.2012.07.002. 22776748

[ref36] Patel G. K. The Role of Nutrition in the Management of Lower Extremity Wounds. Int. J. Lower Extrem. Wounds 2005, 4, 12–22. 10.1177/1534734605274574. 15860449

[ref37] Contardi M.; Lenzuni M.; Fiorentini F.; Summa M.; Bertorelli R.; Suarato G.; Athanassiou A. Hydroxycinnamic Acids and Derivatives Formulations for Skin Damages and Disorders: A Review. Pharmaceutics 2021, 13, 999 10.3390/pharmaceutics13070999. 34371691 PMC8309026

[ref38] Linster C. L.; Van Schaftingen E. Vitamin C: Biosynthesis, Recycling and Degradation in Mammals. FEBS J. 2007, 274, 1–22. 10.1111/j.1742-4658.2006.05607.x. 17222174

[ref39] Michalak M. Plant-Derived Antioxidants: Significance in Skin Health and the Ageing Process. Int. J. Mol. Sci. 2022, 23, 585 10.3390/ijms23020585. 35054770 PMC8776015

[ref40] Calzado-Delgado M.; Guerrero-Pérez M. O.; Yeung K. L. Dissolvable Topical Formulations for Burst and Constant Delivery of Vitamin C. ACS Omega 2023, 8, 12636–12643. 10.1021/acsomega.2c06738. 37065060 PMC10099438

[ref41] Fathi-Azarbayjani A.; Qun L.; Chan Y. W.; Chan S. Y. Novel Vitamin and Gold-Loaded Nanofiber Facial Mask for Topical Delivery. AAPS PharmSciTech 2010, 11, 1164–1170. 10.1208/s12249-010-9475-z. 20661676 PMC2974145

[ref42] Anderson B. Nutrition and Wound Healing: The Necessity of Assessment. Br. J. Nurs. 2005, 14, 30–38. 10.12968/bjon.2005.14.sup5.19955. 16301919

[ref43] Lima C. C.; Pereira A. P. C.; Silva J. R. F.; Oliveira L. S.; Resck M. C. C.; Grechi C. O.; Bernardes M. T. C. P.; Olímpio F. M. P.; Santos A. M. M.; Incerpi E. K.; Garcia J. A. D. Ascorbic Acid for the Healing of Skin Wounds in Rats. Braz. J. Biol. 2009, 69, 1195–1201. 10.1590/s1519-69842009000500026. 19967193

[ref44] Yun I. S.; Yoo H. S.; Kim Y. O.; Rah D. K. Improved Scar Appearance with Combined Use of Silicone Gel and Vitamin C for Asian Patients: A Comparative Case Series. Aesthetic Plast. Surg. 2013, 37, 1176–1181. 10.1007/s00266-013-0210-5. 24091488

[ref45] Pielesz A.; Biniaś D.; Bobiński R.; Sarna E.; Paluch J.; Waksmańska W. The Role of Topically Applied L-Ascorbic Acid in Ex-Vivo Examination of Burn-Injured Human Skin. Spectrochim. Acta, Part A 2017, 185, 279–285. 10.1016/j.saa.2017.05.055. 28591686

[ref46] Moradkhannejhad L.; Abdouss M.; Nikfarjam N.; Mazinani S.; Heydari V. Electrospinning of Zein/Propolis Nanofibers; Antimicrobial Properties and Morphology Investigation. J. Mater. Sci.: Mater. Med. 2018, 29, 165 10.1007/s10856-018-6174-x. 30392146

[ref47] Yao C.; Li X.; Song T. Electrospinning and crosslinking of zein nanofiber mats. Appl. Polym. Sci. 2007, 103, 380–385. 10.1002/app.

[ref48] Miyoshi T.; Toyohara K.; Minematsu H. Preparation of Ultrafine Fibrous Zein Membranes via Electrospinning. Polym. Int. 2005, 54, 1187–1190. 10.1002/pi.1829.

[ref49] Theocharis A. D.; Skandalis S. S.; Gialeli C.; Karamanos N. K. Extracellular Matrix Structure. Adv. Drug Delivery Rev. 2016, 97, 4–27. 10.1016/j.addr.2015.11.001. 26562801

[ref50] Erisken C.; Zhang X.; Moffat K. L.; Levine W. N.; Lu H. H. Scaffold Fiber Diameter Regulates Human Tendon Fibroblast Growth and Differentiation. Tissue Eng., Part A 2013, 19, 519–528. 10.1089/ten.tea.2012.0072. 23150905 PMC3542879

[ref51] Gillgren T.; Barker S. A.; Belton P. S.; Georget D. M. R.; Stading M. Plasticization of Zein: A Thermomechanical, FTIR, and Dielectric Study. Biomacromolecules 2009, 10, 1135–1139. 10.1021/bm801374q. 19317398

[ref52] Kuligowski J.; Quintás G.; Esteve-Turrillas F. A.; Garrigues S.; de la Guardia M. On-Line Gel Permeation Chromatography-Attenuated Total Reflectance-Fourier Transform Infrared Determination of Lecithin and Soybean Oil in Dietary Supplements. J. Chromatogr. A 2008, 1185, 71–77. 10.1016/j.chroma.2008.01.048. 18272158

[ref53] Hari K. D.; Garcia C. V.; Shin G. H.; Kim J. T. Improvement of the UV Barrier and Antibacterial Properties of Crosslinked Pectin/Zinc Oxide Bionanocomposite Films. Polymers 2021, 13, 2403 10.3390/polym13152403. 34372009 PMC8347000

[ref54] Yoshimura T.; Sengoku K.; Fujioka R. Pectin-Based Surperabsorbent Hydrogels Crosslinked by Some Chemicals: Synthesis and Characterization. Polym. Bull. 2005, 55, 123–129. 10.1007/s00289-005-0422-1.

[ref55] Hajiali H.; Heredia-Guerrero J. A.; Liakos I.; Athanassiou A.; Mele E. Alginate Nanofibrous Mats with Adjustable Degradation Rate for Regenerative Medicine. Biomacromolecules 2015, 16, 936–943. 10.1021/bm501834m. 25658494

[ref56] Tehrani E.; Amiri S. Synthesis and Characterization PVA Electro-Spun Nanofibers Containing Encapsulated Vitamin C in Chitosan Microspheres. J. Text. Inst. 2022, 113, 212–223. 10.1080/00405000.2020.1869405.

[ref57] Moores J. Vitamin C: A Wound Healing Perspective. Br. J. Community Nurs. 2013, 18, S6–S11. 10.12968/bjcn.2013.18.Sup12.S6. 24796079

[ref58] Lenzuni M.; Suarato G.; Miele D.; Carzino R.; Ruggeri M.; Bertorelli R.; Sandri G.; Athanassiou A. Development of Biodegradable Zein-Based Bilayer Coatings for Drug-Eluting Stents. RSC Adv. 2021, 11, 24345–24358. 10.1039/d1ra03748j. 35479013 PMC9036829

[ref59] Wang H. J.; Gong S. J.; Lin Z. X.; Fu J. X.; Xue S. T.; Huang J. C.; Wang J. Y. In vivo biocompatibility and mechanical properties of porous zein scaffolds. Biomaterials 2007, 28, 3952–3964. 10.1016/j.biomaterials.2007.05.017. 17582490

[ref60] McCarty S. M.; Percival S. L. Proteases and Delayed Wound Healing. Adv. Wound Care 2013, 2, 438–447. 10.1089/wound.2012.0370. PMC3842891 24688830

[ref61] Pignatelli C.; Perotto G.; Nardini M.; Cancedda R.; Mastrogiacomo M.; Athanassiou A. Electrospun Silk Fibroin Fibers for Storage and Controlled Release of Human Platelet Lysate. Acta Biomater. 2018, 73, 365–376. 10.1016/j.actbio.2018.04.025. 29673841

[ref62] Vogt L.; Liverani L.; Roether J. A.; Boccaccini A. R. Electrospun Zein Fibers Incorporating Poly(Glycerol Sebacate) for Soft Tissue Engineering. Nanomaterials 2018, 8, 150 10.3390/nano8030150. 29518041 PMC5869641

[ref63] Contardi M.; Kossyvaki D.; Picone P.; Summa M.; Guo X.; Heredia-Guerrero J. A.; Giacomazza D.; Carzino R.; Goldoni L.; Scoponi G.; Rancan F.; Bertorelli R.; Di Carlo M.; Athanassiou A.; Bayer I. S. Electrospun Polyvinylpyrrolidone (PVP) Hydrogels Containing Hydroxycinnamic Acid Derivatives as Potential Wound Dressings. Chem. Eng. J. 2021, 409, 128144 10.1016/j.cej.2020.128144.

[ref64] Wojtasik W.; Czemplik M.; Preisner M.; Dymińska L.; Yuan G.; Szopa J.; Kulma A. Pectin from Transgenic Flax Shives Regulates Extracellular Matrix Remodelling in Human Skin Fibroblasts. Process Biochem. 2017, 55, 187–198. 10.1016/j.procbio.2017.02.001.

[ref65] Akit H.; Collins C.; Fahri F.; Hung A.; D’Souza D.; Leury B.; Dunshea F. Dietary Lecithin Decreases Skeletal Muscle COL1A1 and COL3A1 Gene Expression in Finisher Gilts. Animals 2016, 6, 38 10.3390/ani6060038. 27338483 PMC4929418

[ref66] Maji S.; Panda S.; Samal S. K.; Shriwas O.; Rath R.; Pellecchia M.; Emdad L.; Das S. K.; Fisher P. B.; Dash R. Bcl-2 Antiapoptotic Family Proteins and Chemoresistance in Cancer. Advances in Cancer Research, 1st ed.; Elsevier Inc., 2018; Vol. 137. 10.1016/bs.acr.2017.11.001 29405977

[ref67] Zhang L.; Yu J.; Park B. H.; Kinzler K. W.; Vogelstein B. Role of BAX in the Apoptotic Response to Anticancer Agents. Science 2000, 290, 989–992. 10.1126/science.290.5493.989. 11062132

