# Supplementary material for: Plant-Based, Hydrogel-like Microfibers as an Antioxidant Platform for Skin Burn Healing
Source: ACS Appl Bio Mater. 2023 Jul 26;6(8):3103–16. doi: 10.1021/acsabm.3c00214 (PMC10445266; doi:10.1021/acsabm.3c00214)
Supplement: Supplementary file 1 — mt3c00214_si_001.pdf [file mt3c00214_si_001.pdf]

# Plant-based, hydrogel-like microfibers as antioxidant platform for skin burn healing

Fabrizio Fiorentini[a,b,*], Giulia Suarato[a,c,§,*], Maria Summa[c], Dalila Miele[d], Giuseppina Sandri[d], Rosalia Bertorelli[c], Athanassia Athanassiou[a,*]

[a] *Smart Materials Group, Istituto Italiano di Tecnologia, Via Morego 30, Genova 16163, Italy*

[b] *DIBRIS, Università di Genova, Via Opera Pia 13, 16145, Italy*

[c] *Translational Pharmacology, Istituto Italiano di Tecnologia, Via Morego 30, Genova 16163, Italy*

[d] *Department of Drug Science, Università di Pavia, Via Taramelli 12, Pavia 27100, Italy*

[§] *Present address: Institute of Electronics, Information Engineering and Telecommunications (IEIIT), National Research Council of Italy (CNR), Turin, Italy*

** Corresponding authors: fabrizio.fiorentini@iit.it, giulia.suarato@ieiit.cnr.it, athanassia.athanassiou@iit.it*

# Supporting Information

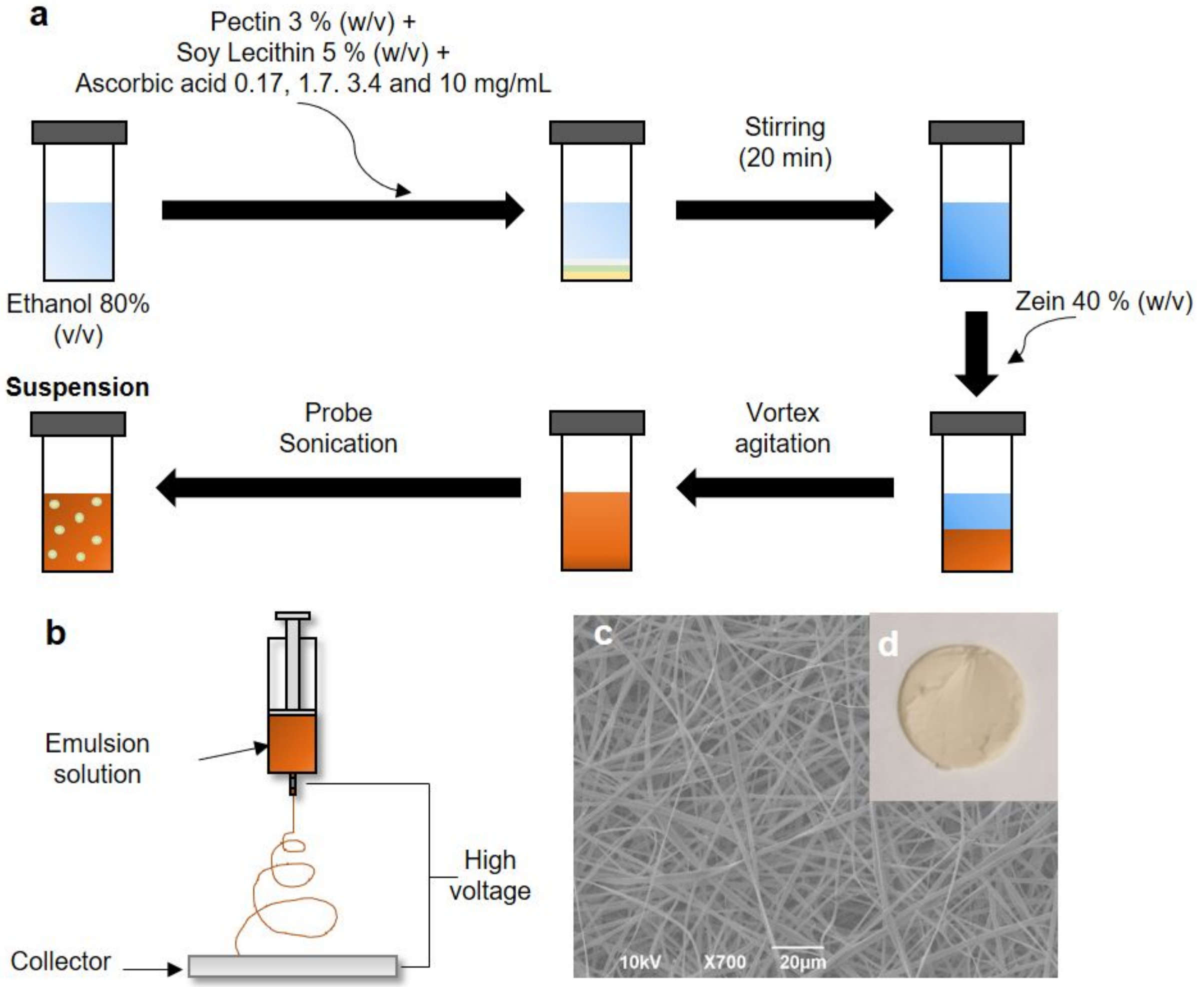


**Figure S1**. **Composite fibers fabrication.** (a) Schematic representation of the procedure used to obtain the suspension solution. (b) Scheme of the vertical electrospinning process. (c) SEM image of the microfibrous mat and (d) macroscopic photo of the final product.

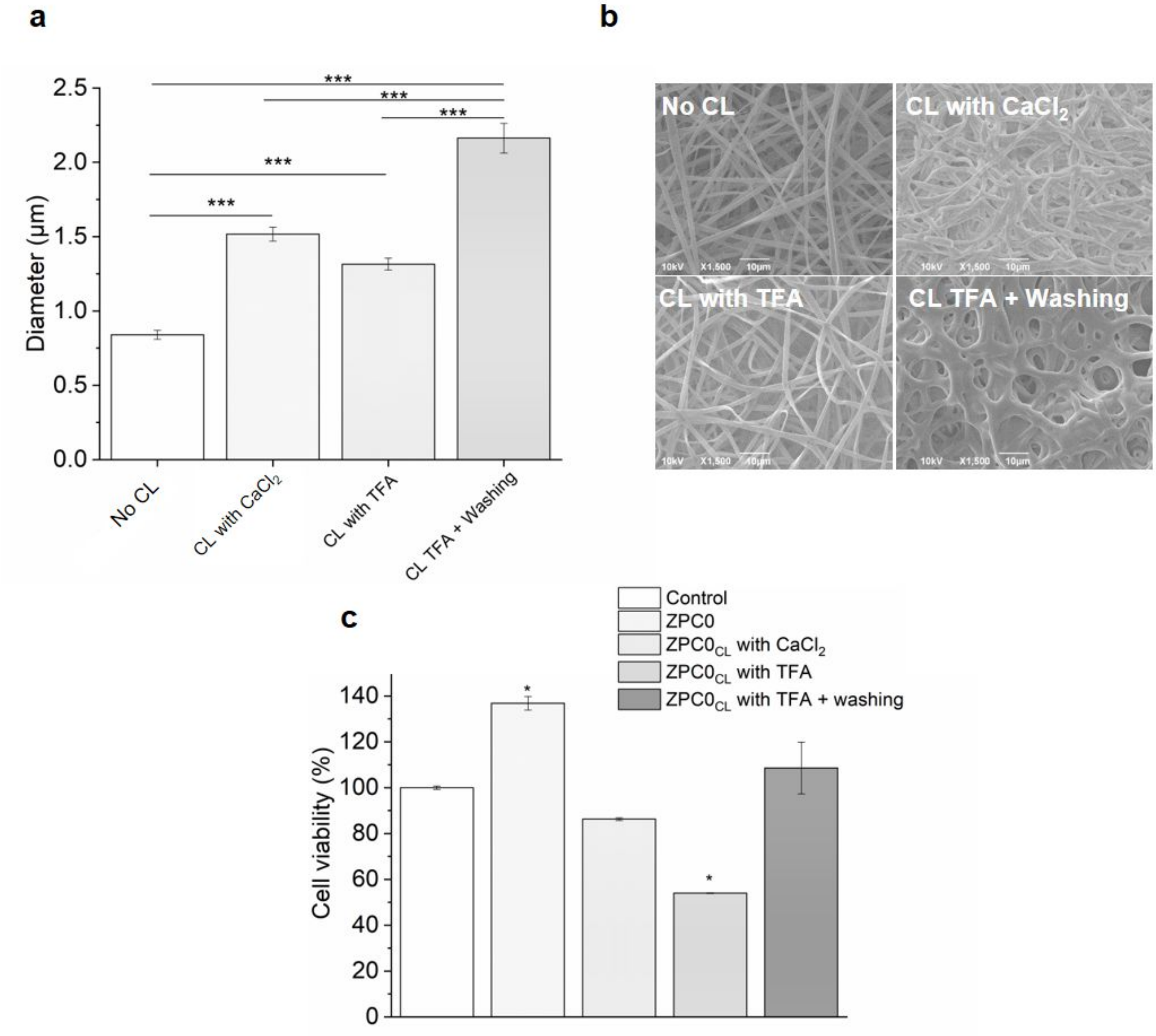


**Figure S2**. **Crosslinking strategy optimization.** (a) Analysis of the microfibers diameter performed on a sample of 100 individual microfibers. (b) SEM images of the microfibers after the different treatments of crosslinking. (c) Cell viability assay on HDFa cells after 24 h of growth in the presence of the extraction media obtained from the microfibers crosslinked with the various approaches. Crosslinking with TFA resulted either in a significant decrease of cell viability, or in a loss of the fibrous architecture when TFA exposition was followed by washing in PBS. Asterisks represent statistical significance (* $p < 0.05$, *** $p < 0.001$).

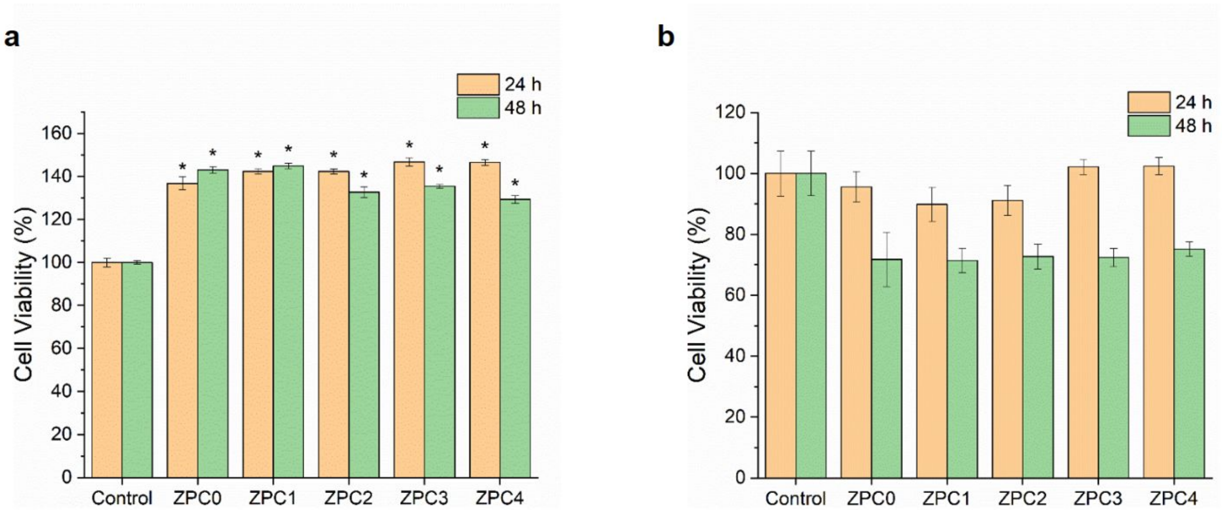


**Figure S3**. ***In vitro* biocompatibility of the composite fibers extracts from non-CL microfibers.** Cell viability assay (a) on HDFa cells and (b) on HaCaT cells after 24 and 48 h of cell growth in the presence of the extracts obtained from the non-CL samples. Asterisks represent statistical significance with respect to the control, untreated samples (* $p < 0.05$).

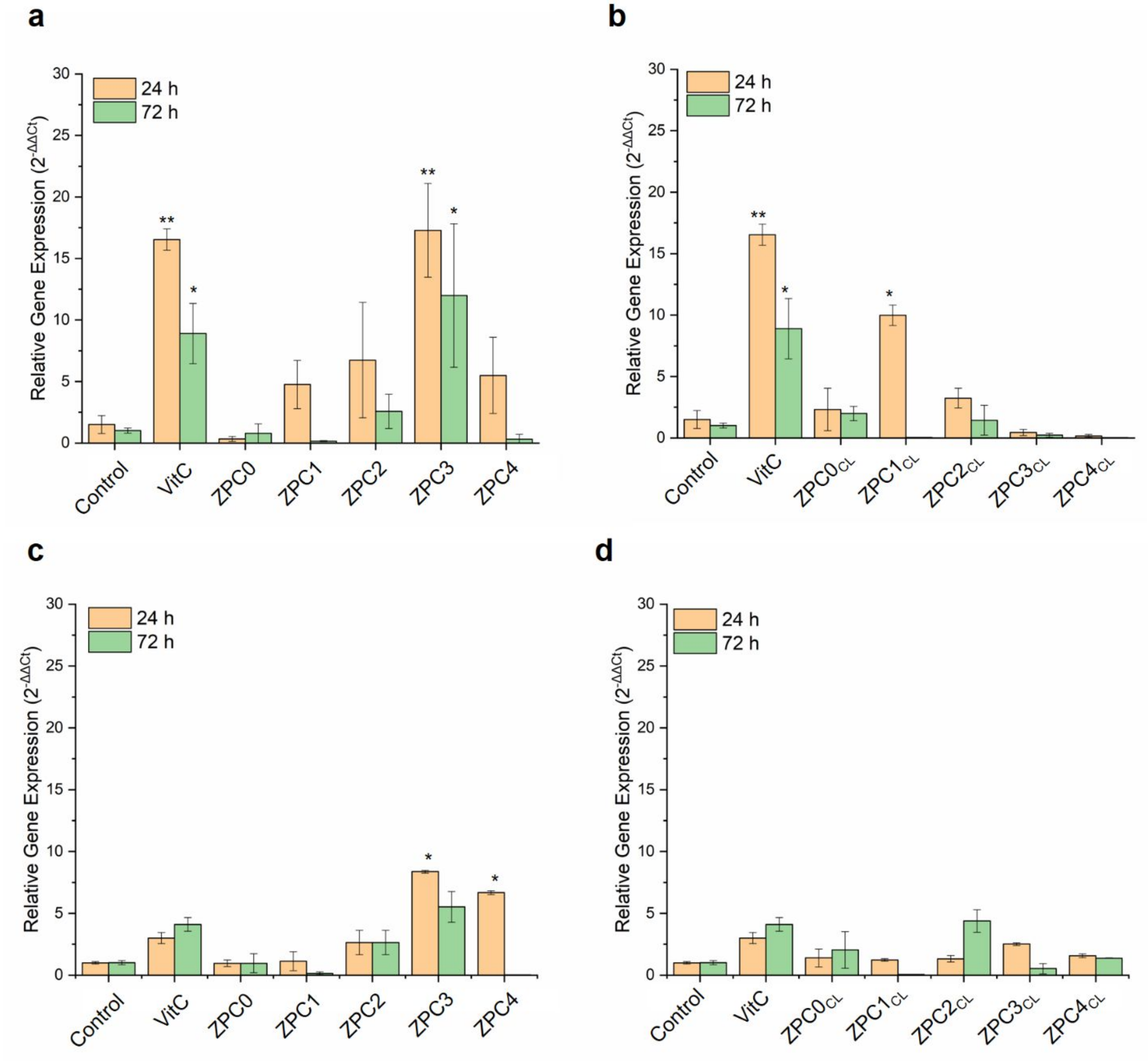


**Figure S4. mRNA quantification analysis onto treated fibroblasts.** *bcl-2* gene expression with real-time PCR on the mRNA extracted from HDFa cells after 1 and 3 days of growth in presence of the extraction medium obtained from (a) non-crosslinked and (b) crosslinked microfibers. *bax* gene expression with real-time PCR on the mRNA extracted from HDFa cells after 1 and 3 days of growth in presence of extraction medium obtained from (c) non-crosslinked and (d) crosslinked microfibers. Asterisks represent statistical significance with respect to the control, untreated samples (* $p < 0.05$, ** $p < 0.005$).
